# Supplementary material for: Whole genome sequencing reveals hidden transmission of carbapenemase-producing Enterobacterales
Source: Nat Commun. 2022 Jun 1;13:3052. doi: 10.1038/s41467-022-30637-5 (PMC9160272; doi:10.1038/s41467-022-30637-5)
Supplement: Supplementary file 1 — Supplementary Information [file 41467_2022_30637_MOESM1_ESM.pdf]

Supplementary Information

Supplementary Methods **2**

Hospital characteristics and infection prevention measures 2

Bacterial isolates and microbiological methods 2

Whole-genome short-read sequencing and assembly 2

Long-read sequencing and hybrid assembly with short-read sequencing data 2

Genomic species identification 2

Carbapenemase gene identification 3

Grouping of isolates into ST-clusters 3

Selection of reference sequence for each ST-cluster from ONT-based hybrid assemblies 3

Bacterial core genome analysis 4

Determination of mutation rate of bacterial species and single-nucleotide polymorphism (SNP) threshold for clonal transmission 4

Establishment of the plasmid genome sequence reference database 5

Identification of carbapenemase-encoding plasmid and determination of plasmid-mediated transmission 5

Supplementary Figures **7**

Supplementary Figure 1. Flowchart describing the grouping of isolates into ST-clusters for bacterial core genome analysis. 7

Supplementary Figure 2. Flowchart describing the core genome analysis 8

Supplementary Tables **9**

Supplementary Table 1. CPE infection prevention and control measures in the participating sites during the study period. 9

Supplementary Table 2. Reference genome sequences selected for each ST-cluster from ONT-based hybrid assemblies. 11

Supplementary Table 3. Mutation rate estimation using BEAST. 13

Supplementary Table 4. Number of plasmid genome sequences forming the carbapenemase gene-specific plasmid databases 14

Supplementary Table 5. Peaks in time-trends analysis (Figure 2) derived from bootstrapping. 15

Supplementary Table 6. Rate of increase and decline of different transmission types. 16

Supplementary Table 7. Factors associated with clonal transmission of CPE for sensitivity analyses restricted to only source isolates which were sampled at least 7 days before genomically-linked acquisition isolates. 17

Supplementary Table 8. Factors associated with plasmid-mediated transmission of CPE for sensitivity analyses restricted to only source isolates which were sampled at least 7 days before genomically-linked acquisition isolates. 19

Supplementary Information on Custom Code **21**

(A) Finding suitable source patients for acquisition patients 21

(B) Determining epidemiologic relationships for source-acquisition pairs 26

(C) Selection of controls 35

References **43**

Supplementary Methods

Hospital characteristics and infection prevention measures

Hospital characteristics and infection prevention measures are described in detail in Figure 3 (in the Manuscript) and Supplementary Table 1.

Bacterial isolates and microbiological methods

Within individual hospital microbiology laboratories, carbapenem non-susceptibility was suspected when minimum inhibitory concentration (MIC) using automated testing systems (VITEK® 2 instrument) was >1 mg/L for imipenem or meropenem, or zone diameter on disc diffusion testing was ≤ 23 mm for imipenem or meropenem disk[1]. Carbapenem MICs were confirmed by Etest (bioMérieux, Marcy l'Étoile, France) with susceptibility defined according to either European Committee on Antimicrobial Susceptibility Testing (EUCAST) or Clinical & Laboratory Standards Institute (CLSI) breakpoints as per the preferred standard adopted by different hospital laboratories. Bacterial isolates were identified using matrix-assisted laser desorption/ionization time-of-flight mass spectrometry (MALDI-TOF MS) (Bruker Daltonics GmbH, Bremen, Germany).

At the National Public Health Laboratory (NPHL), the presence of carbapenemase production was investigated phenotypically using diagnostic meropenem tablets of the KPC/Metallo-β-lactamase Confirmation Kit (Rosco Diagnostica A/S, Taastrup, Denmark). Characterisation of β-lactamase genes was performed by PCR assays targeting class A carbapenemases (bla_{KPC}, bla_{GES}, $bla_{\mathrm{IMI\text{-}1}}$ and $bla_{\mathrm{NMC\text{-}A}}$), class B metallo-β-lactamases (bla_{NDM}, bla_{VIM} and bla_{IMP}) and class D carbapenemases ($bla_{\mathrm{OXA\text{-}48}}$-like and $bla_{\mathrm{OXA\text{-}23}}$ carbapenemases)[1–3]. Carbapenem non-susceptibility due to porin loss associated with extended-spectrum β-lactamases and AmpC overproduction was not investigated. Isolates were stored and re-cultured prior to DNA extraction.

Whole-genome short-read sequencing and assembly

Sequencing libraries for each isolate were prepared according to the manufacturer's recommendation using the Illumina Nextera XT kit, and sequenced on the MiSeq platform as previously described[4].

Adapter and quality trimming were performed using Cutadapt (v1.14), with minimum quality score set to 20, and filtering reads shorter than 20bp[5]. *De novo* assembly of the Illumina reads was performed using SPAdes Genome Assembler (v3.11.1; with the "careful" option activated)[6].

Long-read sequencing and hybrid assembly with short-read sequencing data

Long-read sequencing data was available for 874 isolates using the Oxford Nanopore Technologies (ONT) platform. Libraries for long-read sequencing were prepared using the ONT Rapid Barcoding Kit (RBK004) and sequenced on R9.4.1 flowcells on a GridION platform. Hybrid assemblies of ONT long-read sequence data with Illumina short-read data were generated using the Unicycler (v0.48) with default parameters[7].

Genomic species identification

Bacterial species and sequence-type (ST) were assigned for each isolate based on the genomic sequence using MLST 2.8[8]. For isolates that could not be assigned a species by MLST 2.8, the CGE Bacterial Analysis Pipeline and Kraken (v1.0) (with default parameters) were applied to reattempt species assignment[9,10]. Isolates with species discordance comparing phenotypic with genotypic species assignment on all three methods were excluded from further analysis.

Carbapenemase gene identification

Genomic carbapenemase gene allele assignments were obtained using BLASTN (v2.2.31+) against the NCBI Beta-Lactamase Database (downloaded in May 2017), filtering for 100% identity and 100% subject alignment coverage[11]. For isolates that could not be assigned a carbapenemase gene allele based on the NCBI Beta-Lactamase Database, the CGE Bacterial Analysis Pipeline and SRST2 (v0.2.0) querying the ARG-ANNOT database (ARG-ANNOT-Nt-V3 curated within the SRST2 package) were used[9,12,13]. Isolates for which the genomically-assigned carbapenemase gene on all three methods was discordant compared with the laboratory PCR-determined carbapenemase gene were excluded from further analysis.

Grouping of isolates into ST-clusters

For species with available sequence-type (ST) databases, the species-specific MLST allelic profiles were downloaded from PubMLST (https://pubmlst.org/data/; Date of download: 10 January 2018 for *K. pneumoniae*, 13 March 2018 for *E. coli* and *E. cloacae*, 17 August 2018 for *C. freundii* and *K. oxytoca*). To achieve computational tractability, species-specific sequence-type clusters (ST-clusters) were determined using the PubMLST reference ST profiles, combined with novel STs with non-ambiguous alleles that were detected in our sequenced dataset. This combined dataset was clustered according to the seven gene alleles, allowing for up to two allele mismatches between members of a cluster. STs within an ST-cluster were defined as having ≤2 allele mismatches compared with at least one member of the same ST-cluster.

The isolates from *K. pneumoniae*, *E. coli* and *E. cloacae* within our dataset (accounting for 90% of isolates) were then assigned to an ST-cluster according to the assignments established above (Supplementary Figure 1). Clustering of STs did not result in grouping of isolates for *C. freundii* and *K. oxytoca*.

For each ST-cluster, a complete reference genome sequence was selected by BLASTN to aid in further merging of ST-clusters as follows: The longest assembled contig of a random isolate from each ST-cluster was BLASTN-searched against the NCBI RefSeq Genome Database (https://www.ncbi.nlm.nih.gov/refseq/; date of download: July 2018; filtered for the respective organism name; BLASTN parameters: max target sequences = 500, expect threshold = 0.001). The complete genome with the highest MaxScore was assigned as the reference ST-cluster specific sequence. For ST-clusters where the longest contig did not return a complete genome match using BLAST, the next longest contigs were sequentially used for the BLASTN-search until a successful match was returned. Finally, groups with identical reference NCBI RefSeq closed-genome sequences selected by this method were merged.

The remaining ungrouped isolates were combined with existing ST-clusters by mapping the adapter-trimmed Illumina reads of the isolate against the selected reference sequences using SMALT with default parameters and assigning the isolate to the ST-cluster with the highest reference genome coverage (ensuring a minimum threshold of 90%) (Supplementary Figure 1). Isolates that remained unassigned to ST-clusters by this method were grouped among themselves with isolates that had ≤2 gene allele mismatches based on the seven genes used for MLST determination, as described above (Supplementary Figure 1). For species with no available ST databases, all isolates of the species were treated as a single group (Supplementary Figure 1).

Selection of reference sequence for each ST-cluster from ONT-based hybrid assemblies

For each ST-cluster of *K. pneumoniae*, *E. coli* and *E. cloacae*, the longest fully circularized hybrid-assembled (Nanopore and Illumina) chromosomal genome from the

predominant ST in the ST-cluster was selected as the reference genome. For ST-clusters without a fully circularized hybrid-assembled chromosomal genome from the predominant ST, the longest fully circularized chromosomal genome among all isolates from the ST-cluster was selected. For ST-clusters that still did not have a reference genome, all isolates in the ST-cluster underwent pairwise SNP comparison with all selected reference genomes for the particular species and the reference genome with the least pairwise SNP difference was selected (refer to Supplementary Table 2 for final list of reference sequences assigned).

C. freundii and *K. oxytoca* only had a fully circularized chromosomal genome for the predominant ST of one ST-cluster respectively, and hence this genome was assigned as the reference sequence for all ST-clusters of the species. For *C. amalonaticus, C.koseri, C. rodentium, E. aerogenes, M. morganii,* and *P. mirabilis,* the longest available fully circularized chromosomal genome was selected as the reference sequence. As C. *farmeri* did not have any fully circularized chromosomal genome, the reference used for *C. freundii* was assigned as the reference sequence for the species (refer to Supplementary Table 2 for final list of reference sequences assigned).

Due to the requirement of the recombination filtering step to have more than 3 sequences, sequences of *C. farmeri* (*N*=2) and *C. rodentium* (*N*=2) were analyzed as a single group, and as described above, the reference used for *C. freundii* was assigned as the reference sequence for this group (refer to Supplementary Table 2 for final grouping and list of reference sequences assigned).

Bacterial core genome analysis

Core genome analysis was performed based on previously published methods[14] (Supplementary Figure 2). Gene annotation was performed for all isolates using Prokka (v1.12)[15]. The annotations (gff files) of all isolates for a given species were then used as input for Roary (v1.007002; with parameters -e --mafft -g 60000 -cd 95; BLASTP identity threshold at the default 95%), to generate a core gene alignment of genes that were present in at least 95% of the isolates of the species[16].

For each ST-cluster, the single-nucleotide polymorphisms (SNPs) based on the core gene alignment were called using SNP-sites (v2.4.1), with the consensus sequence of the alignment as a reference[17]. To validate the alignment-based SNP calls, mapping of the short-read data followed by SNP calling was also performed using Snippy (v3.1; https://github.com/tseemann/snippy). Only SNPs in the core gene alignment that were found to be supported by the mapping-based SNP calling were retained in the alignment, while unvalidated SNPs were masked.

The resulting core gene alignment for each ST-cluster was “padded” with invariant sites in a proportion according to the GC content and length of the selected ONT-based reference genome (Supplementary Table 2). Due to the requirement of the recombination filtering step to have more than 3 sequences, core gene alignments of ST-clusters with ≤3 isolates were merged with other ST-clusters within the same species to form groups with 4 or more sequences for analysis. The longest reference sequence was selected as the reference for the merged ST-cluster (refer to Supplementary Table 2 for final grouping and list of reference sequences assigned). The “padded” alignments were then filtered to remove recombinant regions using Gubbins (v2.3.4)[18].

Determination of mutation rate of bacterial species and single-nucleotide polymorphism (SNP) threshold for clonal transmission

For *K. pneumoniae*, *E. coli, E. cloacae* and *C. freundii*, the recombination-filtered alignment of the ST with the largest number of isolates (*K. pneumoniae* ST14, *E. coli* ST131, *E. cloacae* ST93 and *C. freundii* ST22) was “padded” again as described above to produce an

alignment as input for BEAST (v2.5.2) for estimation of a mutation rate for the species (Supplementary Table 3)[19]. Three independent BEAST runs were performed for each dataset, with the following parameters adapted from a previously published study: site model set as bModelTest, strict clock rate, prior set as "coalescent constant population", and Monte Carlo Markov chain (MCMC) iterations set as 300 million[20,21]. The combined effective sample sizes (ESS) for all parameters was greater than 200. The resulting mutation rates (substitutions/genome/year) were 1.01 for *K. pneumoniae*, 0.33 for *E. coli*, 1.06 for *E. cloacae* and 2.72 for *C. freundii*, which were used as the SNP thresholds for defining clonal transmission (Supplementary Table 3).

Due to the limited number of isolates for bacterial species apart from *K. pneumoniae, E. coli, E. cloacae* and *C. freundii*, the SNP thresholds were applied as follows: SNP threshold for *K. pneumoniae* was used for *K. oxytoca, M. morganii* and *P. mirabilis*, SNP threshold for *E. cloacae* was used for *E. aerogenes,* and SNP threshold for *C. freundii* was used for *C. amalonaticus, C. koseri* and *C. rodentium/*C. *farmeri.*

Clonal transmission was established between the isolates if they shared the same ST-cluster, same carbapenemase gene allele and had pairwise SNP count (based on the recombination-filtered core gene alignments) below the BEAST-derived mutation rate threshold, assuming a Poisson distribution for the accumulation of mutations[22]. All carbapenemase genes were considered for establishing clonal transmission for isolates having co-carriage of more than one carbapenemase gene.

Establishment of the plasmid genome sequence reference database

Reference databases of plasmid genome sequences for each carbapenemase gene allele were generated from complete plasmid genome sequences from NCBI RefSeq and fully circularized plasmid genome sequences obtained from the ONT-based hybrid assemblies, as described below.

Using the search term "plasmid", plasmid sequences were downloaded from the NCBI RefSeq database (Date of download: July 2018). The dataset was then filtered to only include sequences with identifiers that meet the keyword criteria "plasmid" AND "circular" NOT "gene" NOT "partial" NOT "incomplete" NOT "putative". Exact duplicate sequences were removed from the dataset. For each carbapenemase gene, all known alleles were downloaded from the ARG-ANNOT database and aligned against the plasmid sequences in the dataset[13]. Only plasmid sequences with 100% identity and 100% coverage of the carbapenemase gene allele were included in the respective carbapenemase gene allele-specific database (Supplementary Table 4).

In addition, a total of 542 fully circularized plasmid genome sequences generated from 529 of the 874 long-read sequencing hybrid assemblies were added to the reference database, resulting in a total of 938 complete plasmid genome sequences (Supplementary Table 4).

Identification of carbapenemase-encoding plasmid and determination of plasmid-mediated transmission

Plasmid identification was performed for all isolates using PlasmidSeeker (v0.1; 2017-04-21) with default parameters, against the carbapenemase gene allele-specific reference databases described above to obtain candidate plasmids[23]. For each isolate, the carbapenemase gene-containing contig (carrying the carbapenemase gene with at least 99% identity and 90% gene coverage) from the assembly was BLAST-aligned (as query) against the candidate plasmids obtained by PlasmidSeeker. Only candidate plasmids that shared ≥90% k-mers with the isolate (i.e. "%Kmers found(F)" ≥90%) and had carbapenemase gene-containing contig alignment with coverage of ≥90% were considered valid. Plasmid-mediated

transmission was then established between a pair of isolates if they shared at least one valid plasmid.

Plasmid assignment was also performed to assign a specific plasmid to each isolate, according to the following selection criteria consecutively: (i) highest query coverage, (ii) highest k-mer coverage, and (iii) longest plasmid. For isolates that had multiple valid potential plasmid assignments, priority was given to the predominant plasmid that was determined for the given carbapenemase gene (refer to the Supplementary Data for the plasmid assignments for our final dataset).

Supplementary Figures

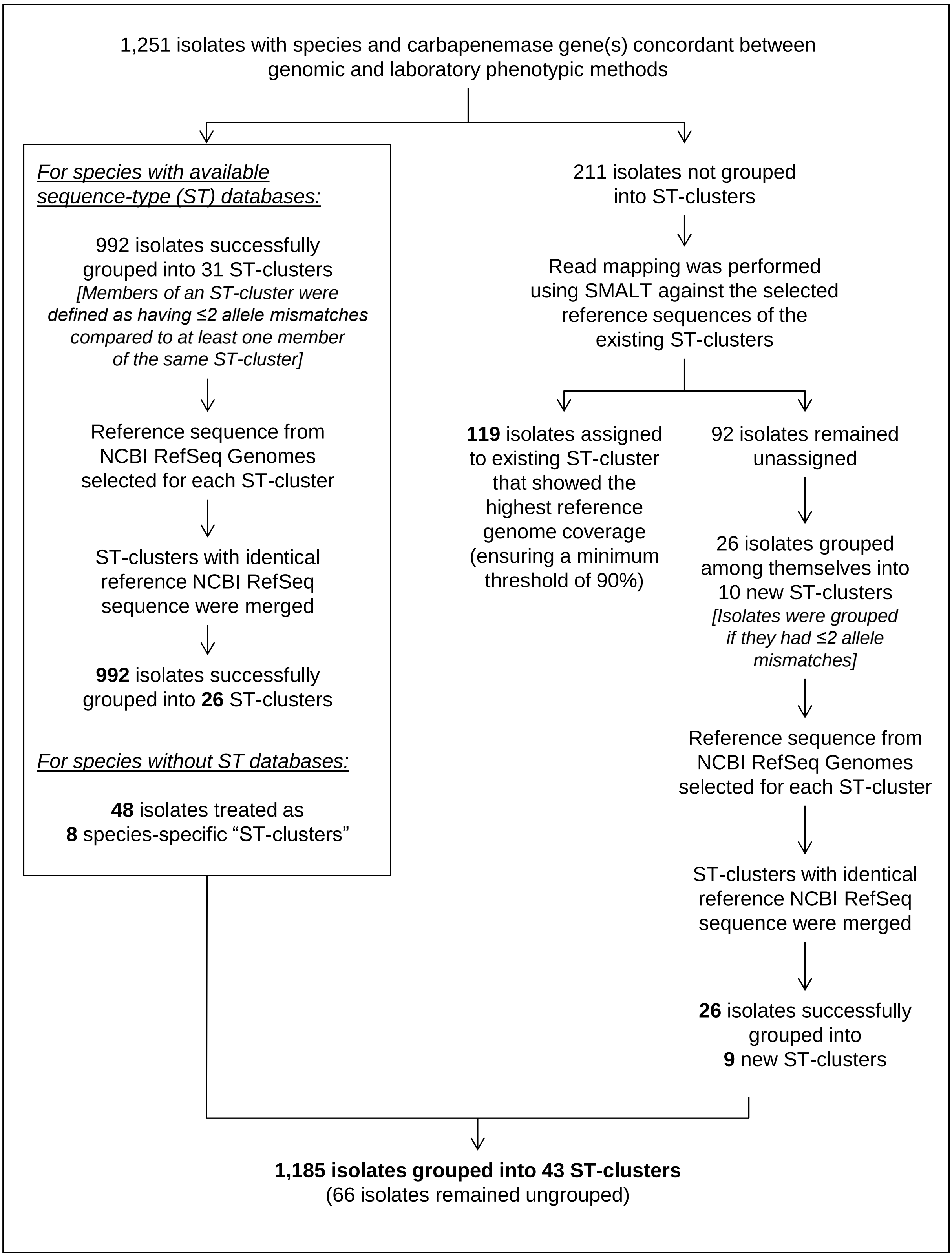

Supplementary Figure 1. Flowchart describing the grouping of isolates into ST-clusters for bacterial core genome analysis.
A total of 1,185 isolates were grouped into 43 ST-clusters.

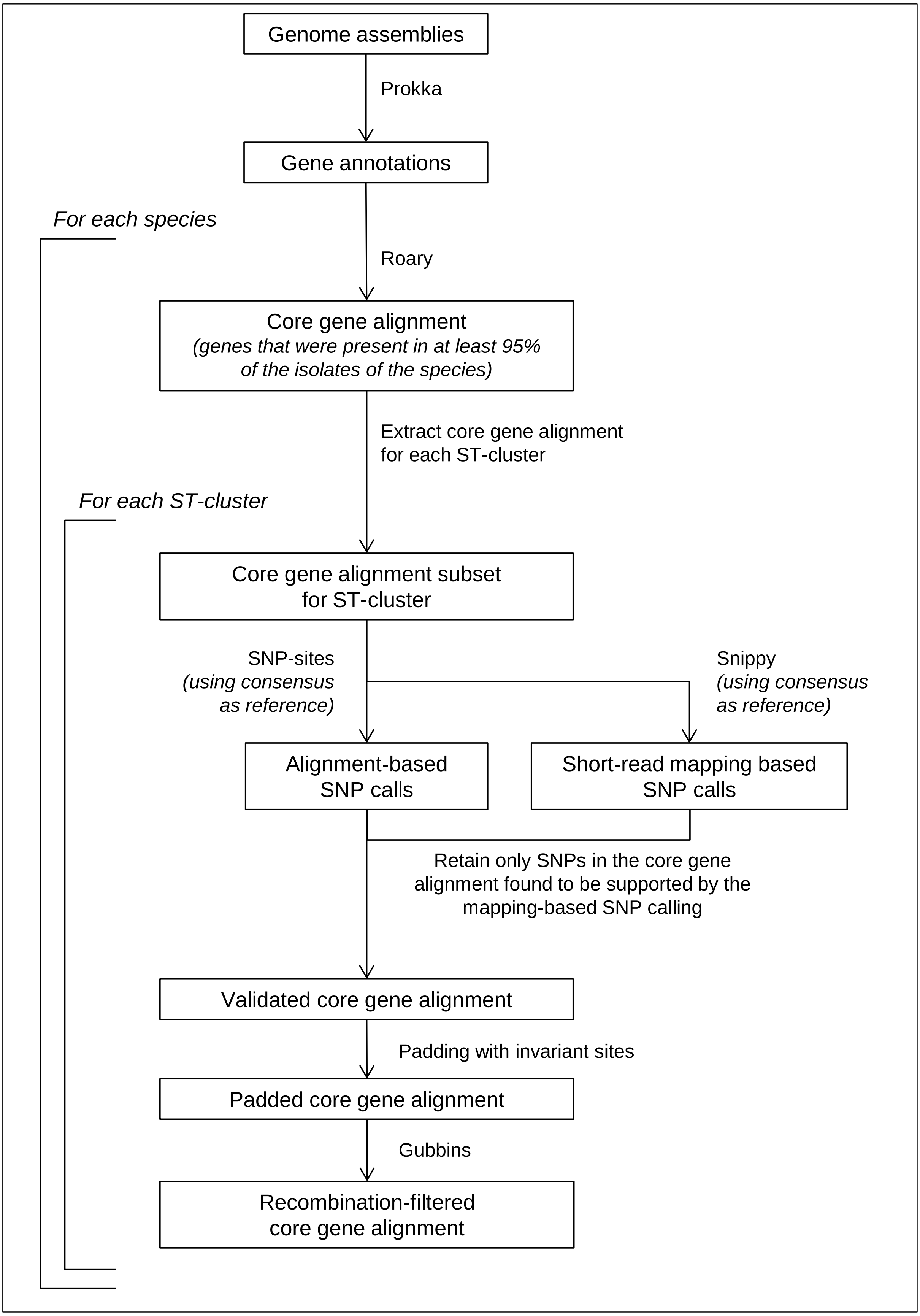

Supplementary Figure 2. Flowchart describing the core genome analysis.

Supplementary Tables

Supplementary Table 1. CPE infection prevention and control measures in the participating sites during the study period.

	Hospital 1	Hospital 2	Hospital 3	Hospital 4	Hospital 5	Hospital 6
Screening of high-risk patients on admission to the hospital	• Since 2011<br>• Single rectal swab<br>• Compliance ~ 20-30%	• Since 2013<br>• Single rectal swab	• Since 2013<br>• Single rectal swab<br>• Based on locally developed risk score	• Since 2013<br>• Two rectal swabs	• Since 2013<br>• Single rectal swabs	• Since 2013<br>• Two rectal swabs<br>• Compliance ~90%
Screening of epidemiologically linked contacts	• Since 2011<br>• Single rectal swab<br>• Compliance ~70-80%	• Since 2013<br>• Single rectal swab	• No	• Since 2013<br>• Two rectal swabs	• Since 2013<br>• Single rectal swabs	• Since 2013<br>• Two rectal swabs<br>• Compliance >90%
Screening on transfer to and from high-risk unit	• No	• Since 2013<br>• Single rectal swab<br>• ICU, Haematology & oncology unit, renal unit	• No	• No	• No	• Yes<br>• Rehabilitation wards
Regular point prevalence survey to estimate burden of CPE	• Yes<br>• CPE prevalence ~1-2%	• No	• No	• No	• No	• No
Single room isolation or cohorting of CPE carriers	• Yes<br>• Compliance to isolation within 24 hours ~90-95%	• Yes<br>• Compliance to isolation within 24 hours >95%	• Yes<br>• Compliance to isolation within 24 hours >95%	• Yes<br>• Compliance to isolation within 24 hours >95%	• Yes<br>• Compliance to isolation within 24 hours >95%	• Yes<br>• Compliance to isolation within 24 hours >95%
Contact precaution (apron/gown and gloves)	• Yes	• Yes	• Yes	• Yes	• Yes	• Yes
Hand hygiene compliance enhancement program	• Yes	• Yes	• Yes	• Yes	• Yes	• Yes

Antimicrobial stewardship program	• Yes	• Yes	• Yes	• Yes	• Yes	• Yes
Environmental hygiene	• Terminal cleaning of rooms occupied by CPE carriers with chlorine-based disinfectant	• Terminal cleaning of rooms occupied by CPE carriers with chlorine-based disinfectant<br>• Hydrogen peroxide vapour since 2015	• Terminal cleaning of rooms occupied by CPE carriers with chlorine-based disinfectant<br>• Hydrogen peroxide vapour since 2012	• Terminal cleaning of rooms occupied by CPE carriers with chlorine-based disinfectant<br>• Hydrogen peroxide vapour or UV light	• Terminal cleaning of rooms occupied by CPE carriers with chlorine-based disinfectant	• Terminal cleaning of rooms occupied by CPE carriers with chlorine-based disinfectant
Surveillance program for endoscopes	• Surveillance cultures since 2012<br>• Culture positive scopes are quarantined and reprocessed until CPE clearance is confirmed	• Surveillance cultures since 2015<br>• Culture positive scopes are quarantined and reprocessed until CPE clearance is confirmed	• Surveillance cultures conducted<br>• Culture positive scopes are quarantined and reprocessed until CPE clearance is confirmed	• No	• No	• Yes<br>• Started since 2012

Abbreviations: CPE, carbapenemase-producing *Enterobacterales*; ICU, intensive care unit

Supplementary Table 2. Reference genome sequences selected for each ST-cluster from ONT-based hybrid assemblies.

Species	ST-cluster	Isolate count	Isolate number of the ONT-based hybrid assembly assigned as reference genome
K. pneumoniae	STcluster3	460	E376
	STcluster7	65	E1190
	STcluster17*	3	E69
	STcluster29	12	E69
	STcluster30*	2	E69
	STcluster31*	3	E69
	Ungrouped	11	
	**Subtotal**	**556**	
E. coli	STcluster1	204	E1006
	STcluster10	95	E883
	STcluster12	27	E826
	STcluster24	4	E1006
	STcluster28	11	E826
	STcluster29	3	E1006
	STcluster4	8	E826
	STcluster42	7	E1006
	STcluster82*	3	E883
	STcluster9	11	E852
	STcluster83*	2	E883
	STcluster84*	2	E883
	Ungrouped	8	
	**Subtotal**	**385**	
E. cloacae	STcluster1	56	E476
	STcluster11	10	E665
	STcluster12*	3	E995
	STcluster18	6	E665
	STcluster23	8	E995
	STcluster24	4	E200
	STcluster3	39	E579
	STcluster6	15	E995
	STcluster25	3	E665
	STcluster27*	2	E995
	STcluster28	3	E665
	STcluster29	5	E665
	Ungrouped	26	
	**Subtotal**	**180**	
C. freundii	STcluster18*	6	E996
	STcluster22	40	E996
	STcluster23*	2	E996
	Ungrouped	19	
	**Subtotal**	**67**	
K. oxytoca	STcluster108*	11	E743
	STcluster135*	2	E743

	Ungrouped	2	
	**Subtotal**	**15**	
C. amalonaticus	-	10	E994
C. farmeri[†]	-	2	E996
C. koseri	-	8	E243
C. rodentium[†]	-	2	E996
E. aerogenes	-	16	E345
M. morganii	-	4	E164
P. mirabilis	-	5	E648
S. marcescens	-	1	
**Total**		**1251**	

[*] These ST-clusters were combined within the species to be analyzed as a single group for filtering of recombination using Gubbins.

[†] *C. farmeri* and *C. rodentium* were analyzed as a single group for filtering of recombination using Gubbins. The reference used for *C. freundii* was assigned as the reference sequence for this group.

Supplementary Table 3. Mutation rate estimation using BEAST.

Species	Isolates used for BEAST*	BEAST meanRate	BEAST 95% HPD interval min.	BEAST 95% HPD interval max. [A]	Length of reference genome (bases) [B]	Mutation rate (substitutions /genome/year) [AxB]
K. pneumoniae	57 isolates from ST14	9.8702 E-8	2.4779 E-8	1.8767 E-7	5,378,540	1.01
E. coli	65 isolates from ST131	2.4685 E-8	7.9668 E-13	6.3444 E-8	5,259,035	0.33
E. cloacae	35 isolates from ST93	1.0178 E-7	1.1773 E-8	2.2133 E-7	4,814,122	1.06
C. freundii	28 isolates from ST22	3.1486 E-7	1.1224 E-7	5.2888 E-7	5,149,685	2.72

* These are from the ST with the largest number of isolates

Supplementary Table 4. Number of plasmid genome sequences forming the carbapenemase gene-specific plasmid databases.

Carbapenemase gene allele	Obtained from NCBI RefSeq	Obtained from ONT-based hybrid assembly	Total
IMP-1	1	0	1
IMP-4	12	4	16
KPC-2	174	103	277
KPC-6	2	0	2
NDM-1	117	310	427
NDM-4	2	5	7
NDM-5	32	18	50
NDM-7	8	18	26
NDM-9	4	4	8
OXA-23	13	0	13
OXA-48	26	21	47
OXA-181	5	52	57
OXA-232	0	6	6
VIM-4	0	1	1
**Total**	**396**	**542**	**938**

Supplementary Table 5. Peaks in time-trends analysis (Figure 2) derived from bootstrapping.

§	Transmission	Type	Peak (YYYY-MM)	Lower (YYYY-MM)	Upper (YYYY-MM)	Significant (Yes/ No)
1	Clonal	Overall	2014-03	2013-12	2014-06	Yes
2	Plasmid-mediated	Overall	2014-11	2014-04	2015-04	No
3	Clonal	Surveillance	2014-04	2014-01	2014-07	Yes
4	Plasmid-mediated	Surveillance	2014-11	2014-05	2015-04	No
5	Clonal	Clinical	2013-06	2013-02	2015-04	No
6	Plasmid-mediated	Clinical	2013-09	2013-03	2015-04	No
7	Clonal	Hospital / indirect	2015-04	2014-05	2015-04	No
8	Clonal	Hospital / direct	2014-02	2013-09	2015-04	No
9	Clonal	Ward / indirect	2014-04	2013-11	2015-04	No
10	Clonal	Ward / direct	2013-12	2013-07	2014-03	Yes
11	Clonal	No overlap	2015-04	2014-03	2015-04	No
12	Plasmid-mediated	Hospital / indirect	2015-04	2013-06	2015-04	No
13	Plasmid-mediated	Hospital / direct	2013-10	2012-11	2015-04	No
14	Plasmid-mediated	Ward / indirect	2015-01	2013-10	2015-04	No
15	Plasmid-mediated	Ward / direct	2014-08	2014-04	2015-04	No
16	Plasmid-mediated	No overlap	2015-04	2013-01	2015-04	No

Supplementary Table 6. Rate of increase and decline of different transmission types.

**Transmission**	**Type**	**Rate of increase (acquisition patients per 10,000 patient-days per month) (95%CI)[*]**	**Rate of decline (acquisition patients per 10,000 patient-days per month) (95%CI)**
Clonal	Overall[†]	0.021 (0.015, 0.027)	–0.026 (–0.049, –0.007)
	Surveillance cultures only[‡]	0.017 (0.011, 0.024)	–0.035 (–0.060, –0.010)
	Clinical cultures only	0.003 (0.002, 0.006)	NA
Plasmid-mediated	Overall	0.016 (0.011, 0.024)	NA
	Surveillance cultures only	0.013 (0.008, 0.019)	NA
	Clinical cultures only	0.003 (0.002, 0.005)	NA
Clonal	Ward, direct[§]	0.008 (0.004, 0.012)	–0.017 (–0.029, –0.005)
	Ward, indirect	0.003 (0.002, 0.006)	NA
	Hospital, direct	0.002 (0.001, 0.003)	NA
	Hospital, indirect	0.002 (0.001, 0.005)	NA
Plasmid-mediated	Ward, direct	0.009 (0.006, 0.015)	NA
	Ward, indirect	0.004 (0.002, 0.005)	NA
	Hospital, direct	0.000 (0.000, 0.001)	NA
	Hospital, indirect	0.001 (0.000, 0.003)	NA

[*] Unless otherwise specified the rate of increase is determined over the study period.

[†] For clonal transmission, the rate of increase was determined from September 2010 to the peak (estimated to be March 2014) and the rate of decrease was determined from then until April 2015.

[‡] For clonal transmission limited to surveillance cultures, the rate of increase was determined from September 2010 to the peak (estimated to be April 2014) and the rate of decrease was determined from then until April 2015.

[§] For ward-direct-bacteria-mediated transmission, the rate of increase was determined from September 2010 to the peak (estimated to be December 2013) and the rate of decrease was determined from then until April 2015.

Supplementary Table 7. Factors associated with clonal transmission of CPE for sensitivity analyses restricted to only source isolates which were sampled at least 7 days before genomically-linked acquisition isolates.

Variable	Clonal transmission case pairs (*N*=1198) (%)	Control pairs (*N*=1198) (%)	Clonal transmission case pairs* (weighted %)	Control pairs* (weighted %)	Univariable analysis†		Multivariable analysis†	
					OR (95%CI)	P-value	aOR (95%CI)	P-value
**Hospital or ward contact**								
No hospital nor ward contact	274	638	22.5%	49.4%	ref	–	–	–
Direct ward contact	59	8	9.2%	0.6%	6.99 (4.17–11.7)	<0.0001	5.08 (2.82–9.15)	<0.0001
Indirect ward contact	252	139	22.3%	11.2%	3.80 (2.56–5.64)	<0.0001	3.23 (2.15–4.86)	<0.0001
Direct hospital contact (No ward contact)	225	80	21.0%	9.6%	3.97 (2.51–6.28)	<0.0001	3.83 (2.39–6.14)	<0.0001
Indirect hospital contact (No ward contact)	388	333	25.0%	29.2%	2.23 (1.46–3.41)	0.0002	2.08 (1.35–3.20)	0.0008
**Discipline contact**								
No contact	911	1040	74.7%	87.3%	ref	–	–	–
Direct contact	79	23	9.2%	2.1%	2.59 (1.63–4.13)	<0.0001	1.27 (0.79–2.03)	0.32
Indirect contact	208	135	16.1%	10.6%	1.56 (1.10–2.20)	0.012	1.12 (0.79–1.57)	0.53
**Procedure contact**								
No contact	1185	1185	99.5%	99.3%	ref	–	–	–
Direct contact	0	0	0.0%	0.0%	–	–	–	–
Indirect contact	13	13	0.5%	0.7%	0.86 (0.24–3.06)	0.82	–	–

**Bacterial species**								
Escherichia coli	217	493	15.7%	41.1%	ref	–	–	–
Klebsiella pneumoniae	662	298	60.0%	25.9%	4.25 (2.64–6.84)	<0.0001	4.10 (2.54–6.62)	<0.0001
Enterobacter spp	284	278	18.3%	23.7%	1.78 (1.03–3.07)	0.038	2.01 (1.19–3.39)	0.0095
Others	35	129	6.0%	9.3%	1.42 (0.68–2.93)	0.35	1.27 (0.61–2.65)	0.52
**Genotypes**								
bla_{KPC}	380	484	45.7%	45.3%	ref	–	–	–
bla_{NDM}	509	550	33.7%	41.3%	1.24 (0.87–1.77)	0.23	1.32 (0.85–2.06)	0.22
$bla_{OXA\text{-}type}$	287	95	16.3%	8.0%	2.13 (1.28–3.53)	0.0034	2.14 (1.18–3.89)	0.013
Others	22	69	4.3%	5.4%	0.96 (0.43–2.17)	0.92	0.93 (0.38–2.28)	0.87
**Community contact**[‡]								
No contact	1197	1198	–	–	–	–	–	–
Same household	0	0	–	–	–	–	–	–
Same zipcode	1	0	–	–	–	–	–	–

Abbreviations: OR, odds-ratio; aOR, adjusted odds-ratio; ref, reference; –, not applicable

[*] To correct for potential bias from clustering, the prevalence of epidemiologic risk factors were inversely-weighted by a factor of one over the number of case-control pairs with identical source patient and by reducing the sample size to derive standard errors concomitantly.

[†] Univariable and multivariable analyses were conducted based on the weighted percentages using conditional logistic regression on matched case-control pairs. The Wald chi-square test was performed for all the risk factors with an α level of 0.05 (two-sided). No adjustment was made for multiple comparisons.

[‡] Community contact was excluded from univariable and multivariable analyses as the frequency of exposure was too low.

Supplementary Table 8. Factors associated with plasmid-mediated transmission of CPE for sensitivity analyses restricted to only source isolates which were sampled at least 7 days before genomically-linked acquisition isolates.

Variable	Plasmid-mediated transmission case pairs (*N*=32721) (%)	Control pairs (*N*=32721) (%)	Plasmid-mediated transmission case pairs* (weighted %)	Control pairs* (weighted %)	Univariable analysis†		Multivariable analysis†	
					OR (95%CI)	P-value	aOR (95%CI)	P-value
**Hospital or ward contact**								
No hospital nor ward contact	9810	15263	39.4%	52.6%	ref	–	–	–
Direct ward contact	656	245	2.3%	0.6%	2.13 (0.93–4.90)	0.075	2.10 (0.72–6.16)	0.17
Indirect ward contact	6347	4209	16.2%	11.1%	1.52 (0.89–2.61)	0.13	1.48 (0.79–2.76)	0.22
Direct hospital contact (No ward contact)	5031	2319	14.2%	5.8%	1.84 (1.11–3.05)	0.018	1.77 (1.03–3.02)	0.038
Indirect hospital contact (No ward contact)	10877	10685	27.9%	29.9%	1.20 (0.71–2.00)	0.50	1.17 (0.67–2.06)	0.59
**Discipline contact**								
No contact	26450	28509	83.4%	88.2%	ref	–	–	–
Direct contact	1426	612	3.9%	1.7%	1.48 (0.87–2.50)	0.15	0.99 (0.50–1.95)	0.97
Indirect contact	4845	3600	12.8%	10.1%	1.17 (0.70–1.94)	0.55	1.01 (0.60–1.71)	0.97
**Procedure contact**								
No contact	32440	32449	99.4%	99.4%	ref	–	–	–
Direct contact	6	0						
Indirect contact	275	272	0.5%	0.6%	0.99 (0.38–2.63)	0.99	0.83 (0.34–2.06)	0.69

**Genotypes**								
bla$_{KPC}$	17437	8688	51.0%	48.8%	ref	–		
bla$_{NDM}$	14312	16479	40.2%	30.9%	0.90 (0.58–1.38)	0.63	0.95 (0.62–1.47)	0.83
bla$_{OXA\text{-}type}$	311	5113	6.2%	12.9%	0.50 (0.14–1.76)	0.28	0.53 (0.16–1.78)	0.31
Others	661	2441	2.6%	7.4%	0.38 (0.06–2.53)	0.32	0.40 (0.06–2.64)	0.34
**Community contact**‡								
No contact	32710	32714	–	–	–	–	–	–
Same household	1	0	–	–	–	–	–	–
Same zipcode	10	7	–	–	–	–	–	–

Abbreviations: OR, odds-ratio; aOR, adjusted odds-ratio; ref, reference; –, not applicable

* To correct for potential bias from clustering, the prevalence of epidemiologic risk factors were inversely-weighted by a factor of one over the number of case-control pairs with identical source patient and by reducing the sample size to derive standard errors concomitantly..

† Univariable and multivariable analyses were conducted based on the weighted percentages using conditional logistic regression on matched case-control pairs. The Wald chi-square test was performed for all the risk factors with an α level of 0.05 (two-sided). No adjustment was made for multiple comparisons.

‡ Community contact was excluded from univariable and multivariable analyses as the frequency of exposure was too low.

Supplementary Information on Custom Code

We provide here a description of methods that utilize custom code, with pseudocode where applicable. The custom code can be accessed on Github at https://github.com/nataschamay/cp_transmission_2021 as well as on Zenodo at https://doi.org/10.5281/zenodo.6363989. Demo input and output files are also provided.
Separately, the statistical programming code is provided as a zipped package under Supplementary Software.

(A) Finding suitable source patients for acquisition patients

After grouping of isolates into ST-clusters, performing core genome analysis and determination of mutation rate of bacterial species and single-nucleotide polymorphism (SNP) threshold for clonal transmission (as described in the Supplementary Appendix), we have the following:
(1) List of all isolates and their corresponding ST cluster

- Already excluded isolates that (i) genomically-assigned species and/or CP gene were discordant compared with the laboratory-determined species and/or CP gene, (ii) lack date of culture, (iii) lack patient admission data

(2) Pairs of isolates that meet the SNP threshold for clonal transmission

- Clonal transmission was established between the isolates if they shared the same ST-cluster, same CP gene allele and had pairwise SNP count (based on the recombination-filtered core gene alignments) below the BEAST-derived mutation rate threshold, assuming a Poisson distribution for the accumulation of mutations
- The custom code for this step is **dnaDist_Poisson.R** and can be obtained from https://github.com/nataschamay/cp_transmission_2021/tree/main/1_dnaDist

The custom code for finding suitable source patients for acquisition patients is **find_pairs.pl** and can be obtained from https://github.com/nataschamay/cp_transmission_2021/tree/main/2_find_pairs

Pseudocode for find_pairs.pl

```
Foreach ST cluster and CP gene allele{

        Get unique list of all patients who have isolates in this ST group and CP gene allele

        Foreach patient (by ST cluster and CP gene allele){
```

```
        (A1) Select the earliest isolate (by date of culture (DOC)*) per patient per CP gene (see Demo Table 1 for example)
        (An isolate of the same patient, same ST cluster and same CP gene allele but having a later date of culture is filtered
        off. If there are more than one isolate on the earliest date of the same patient, same ST cluster and same CP gene
        allele, only one is retained (random))

        * Dates of culture are formatted as decimals using a Microsoft Excel formula:
        =YEAR(A1)+((A1)-DATE(YEAR(A1),1,0))/(DATE(YEAR(A1)+1,1,0)-DATE(YEAR(A1),1,0))
        6 decimal places were sufficient to differentiate 1 day.

        (A2) For each earliest isolate per patient, search for potential source isolates from other patients (with isolates of
        the same ST cluster and CP gene allele) that have date of culture earlier or equal to the selected earliest isolate of
        this patient

        (A3) For each pair of isolates (source-acquisition) determined in step A2, populate the core genome-based SNPcount and
        Poisson-based transmission status.
        See Demo Table 3 for source-acquisition pairs for which transmission is plausible, i.e. SNP count met the BEAST-derived
        mutation rate threshold)

        Notes:
        a) An isolate that is the earliest for a given patient (hence is the acquisition isolate), can also serve as a source
        isolate to an isolate of a different patient
        b) Isolates with multiple CP genes were allowed to match earlier isolates with any or all of the CP genes
       }
}
```

Further manual filtering steps done using Microsoft Excel:
(B1) Exclude later isolate from same patient if it has the same CP gene regardless of species (but do not exclude if date of culture is the same - to give a chance to both isolates to pair with a source isolate) **(see Demo Table 2 for example)**
(B2) Exclude later isolate from same source patient, to retain only the earliest isolate meeting clonal transmission criteria. Hence, final result will have unique source-acquisition patient pairs **(see Demo Table 3)**

Demo Table 1: Example for the selection of earliest isolate per patient for ST cluster “cfreundii_CCgroup22_40samples” (i.e. the combined outcome of steps A1 and B1 described above).

Note: The Patient ID, Isolate ID and dates stated here are for purpose of illustration and not actual patient information.

Patient ID	Isolate ID	Date of culture	Date of culture (decimal)	ST cluster	Species	ST	CP gene	Retain?
Patient_4	CF1	30/5/2019	2019.410959	cfreundii_CCgroup22_40samples	Citrobacter freundii	107	blaNDM-1	Y
Patient_4	CF2	8/6/2019	2019.435617	cfreundii_CCgroup22_40samples	Citrobacter freundii	107	blaNDM-1	N (Later isolate)
Patient_6	CF3	13/6/2019	2019.449315	cfreundii_CCgroup22_40samples	Citrobacter freundii	22	blaNDM-1	N (Later isolate)*
Patient_6	CF4	25/6/2019	2019.482192	cfreundii_CCgroup22_40samples	Citrobacter freundii	22	blaNDM-1	N (Later isolate)
Patient_12	CF5	23/3/2020	2020.227398	cfreundii_CCgroup22_40samples	Citrobacter freundii	22	blaNDM-1	Y
Patient_1	CF6	11/2/2019	2019.115069	cfreundii_CCgroup22_40samples	Citrobacter freundii	22	blaNDM-1	Y
Patient_42	CF7	8/7/2017	2017.519133	cfreundii_CCgroup22_40samples	Citrobacter freundii	22	blaNDM-1	Y
Patient_30	CF8	23/2/2019	2019.147945	cfreundii_CCgroup22_40samples	Citrobacter freundii	-	blaNDM-1	Y
Patient_3	CF9	14/3/2019	2019.200000	cfreundii_CCgroup22_40samples	Citrobacter freundii	98	blaIMP-1	Y
Patient_51	CF10	26/5/2019	2019.400000	cfreundii_CCgroup22_40samples	Citrobacter freundii	22	blaNDM-1	Y
Patient_58	CF11	6/6/2019	2019.430137	cfreundii_CCgroup22_40samples	Citrobacter freundii	22	blaNDM-1	Y
Patient_7	CF12	25/6/2019	2019.482192	cfreundii_CCgroup22_40samples	Citrobacter freundii	-	blaKPC-2	N (Later isolate)*
Patient_57	CF13	12/3/2017	2017.196729	cfreundii_CCgroup22_40samples	Citrobacter freundii	91	blaIMP-1	Y
Patient_16	CF14	15/9/2019	2019.706850	cfreundii_CCgroup22_40samples	Citrobacter freundii	22	blaNDM-1	Y
Patient_18	CF15	1/10/2019	2019.750685	cfreundii_CCgroup22_40samples	Citrobacter freundii	107	blaNDM-1	Y
Patient_9	CF16	18/10/2019	2019.797261	cfreundii_CCgroup22_40samples	Citrobacter freundii	22	blaOXA-48	N (Later isolate)
Patient_9	CF17	25/10/2019	2019.816439	cfreundii_CCgroup22_40samples	Citrobacter freundii	22	blaOXA-48	N (Later isolate)
Patient_9	CF18	25/10/2019	2019.816439	cfreundii_CCgroup22_40samples	Citrobacter freundii	22	blaOXA-48	N (Later isolate)
Patient_9	CF19	17/1/2020	2020.046576	cfreundii_CCgroup22_40samples	Citrobacter freundii	22	blaOXA-48	N (Later isolate)
Patient_36	CF20	23/3/2020	2020.227398	cfreundii_CCgroup22_40samples	Citrobacter freundii	22	blaNDM-1	Y
Patient_17	CF21	24/9/2017	2017.732248	cfreundii_CCgroup22_40samples	Citrobacter freundii	22	blaNDM-1	Y
Patient_52	CF22	7/11/2017	2017.852467	cfreundii_CCgroup22_40samples	Citrobacter freundii	22	blaNDM-1	Y
Patient_56	CF23	14/4/2018	2018.284932	cfreundii_CCgroup22_40samples	Citrobacter freundii	22	blaNDM-1	Y
Patient_55	CF24	24/9/2018	2018.731507	cfreundii_CCgroup22_40samples	Citrobacter freundii	22	blaNDM-1	Y (keep to allow pairing#)
Patient_48	CF25	18/10/2018	2018.797261	cfreundii_CCgroup22_40samples	Citrobacter freundii	22	blaNDM-1	Y
Patient_43	CF26	25/10/2018	2018.816439	cfreundii_CCgroup22_40samples	Citrobacter freundii	-	blaNDM-1	N (Later isolate)*
Patient_40	CF27	25/10/2018	2018.816439	cfreundii_CCgroup22_40samples	Citrobacter freundii	22	blaNDM-1	Y
Patient_59	CF28	26/10/2018	2018.819178	cfreundii_CCgroup22_40samples	Citrobacter freundii	22	blaNDM-1	Y
Patient_38	CF29	31/10/2018	2018.832877	cfreundii_CCgroup22_40samples	Citrobacter freundii	22	blaNDM-1	Y
Patient_21	CF30	5/11/2018	2018.846576	cfreundii_CCgroup22_40samples	Citrobacter freundii	22	blaNDM-1	Y
Patient_14	CF31	6/11/2018	2018.849315	cfreundii_CCgroup22_40samples	Citrobacter freundii	22	blaNDM-1	Y
Patient_31	CF32	31/12/2018	2019.000000	cfreundii_CCgroup22_40samples	Citrobacter freundii	22	blaNDM-1	Y
Patient_20	CF33	24/1/2019	2019.065754	cfreundii_CCgroup22_40samples	Citrobacter freundii	22	blaNDM-1	Y
Patient_15	CF34	23/12/2017	2017.978150	cfreundii_CCgroup22_40samples	Citrobacter freundii	64	blaKPC-2	Y (keep to allow pairing)
Patient_50	CF35	8/3/2019	2019.183562	cfreundii_CCgroup22_40samples	Citrobacter freundii	22	blaKPC-2	Y
Patient_7	CF36	3/7/2019	2019.504110	cfreundii_CCgroup22_40samples	Citrobacter freundii	-	blaKPC-2	N (Later isolate)
Patient_25	CF37	22/10/2019	2019.808219	cfreundii_CCgroup22_40samples	Citrobacter freundii	112	blaKPC-2	Y (keep to allow pairing)
Patient_11	CF38	19/1/2020	2020.052055	cfreundii_CCgroup22_40samples	Citrobacter freundii	-	blaKPC-2	Y (keep to allow pairing)
Patient_9	CF39	8/10/2019	2019.769863	cfreundii_CCgroup22_40samples	Citrobacter freundii	22	blaNDM-1,blaOXA-48	Y
Patient_24	CF40	20/5/2019	2019.383562	cfreundii_CCgroup22_40samples	Citrobacter freundii	22	blaNDM-1,blaOXA-48	Y

(*) Determined to be “later isolate” due to presence of an earlier isolate from another species (see Demo Table 2)

Demo Table 2: Later isolate from same patient excluded if they have the same CP gene as earlier isolate <u>regardless of species</u>.
Note: The Patient ID, Isolate ID and dates stated here are for purpose of illustration and not actual patient information.

Patient ID	Isolate ID	Date of culture	Date of culture (decimal)	Species	CP gene	Retain?
Patient_4	CF1	30/5/2019	2019.410959	Citrobacter freundii	blaNDM-1	Y
Patient_4	CF2	8/6/2019	2019.435617	Citrobacter freundii	blaNDM-1	N (Later isolate)
Patient_6	ECO1	12/6/2019	2019.446576	Escherichia coli	blaNDM-1	Y
Patient_6	CF3	13/6/2019	2019.449315	Citrobacter freundii	blaNDM-1	N (Later isolate)
Patient_6	CF4	25/6/2019	2019.482192	Citrobacter freundii	blaNDM-1	N (Later isolate)
Patient_7	ECL28	23/6/2019	2019.476713	Enterobacter cloacae	blaKPC-2	Y
Patient_7	KP3	23/6/2019	2019.476713	Klebsiella pneumoniae	blaKPC-2	Y (keep to allow pairing#)
Patient_7	CF12	25/6/2019	2019.482192	Citrobacter freundii	blaKPC-2	N (Later isolate)
Patient_7	CF36	3/7/2019	2019.504110	Citrobacter freundii	blaKPC-2	N (Later isolate)
Patient_9	CF39	8/10/2019	2019.769863	Citrobacter freundii	blaNDM-1,blaOXA-48	Y
Patient_9	CF16	18/10/2019	2019.797261	Citrobacter freundii	blaOXA-48	N (Later isolate)
Patient_9	CF17	25/10/2019	2019.816439	Citrobacter freundii	blaOXA-48	N (Later isolate)
Patient_9	CF18	25/10/2019	2019.816439	Citrobacter freundii	blaOXA-48	N (Later isolate)
Patient_9	CF19	17/1/2020	2020.046576	Citrobacter freundii	blaOXA-48	N (Later isolate)
Patient_11	CA1	19/1/2020	2020.052055	Citrobacter amalonaticus	blaKPC-2	Y
Patient_11	CF38	19/1/2020	2020.052055	Citrobacter freundii	blaKPC-2	Y (keep to allow pairing#)
Patient_15	EA1	23/12/2017	2017.978150	Enterobacter aerogenes	blaKPC-2	Y
Patient_15	CF34	23/12/2017	2017.978150	Citrobacter freundii	blaKPC-2	Y (keep to allow pairing#)
Patient_25	ECL41	22/10/2019	2019.808219	Enterobacter cloacae	blaKPC-2	Y
Patient_25	CF37	22/10/2019	2019.808219	Citrobacter freundii	blaKPC-2	Y (keep to allow pairing#)
Patient_43	KP9	11/10/2018	2018.778082	Klebsiella pneumoniae	blaNDM-1	Y
Patient_43	KP10	25/10/2018	2018.816439	Klebsiella pneumoniae	blaNDM-1	N (Later isolate)
Patient_43	CF26	25/10/2018	2018.816439	Citrobacter freundii	blaNDM-1	N (Later isolate)
Patient_55	ECL42	24/9/2018	2018.731507	Enterobacter cloacae	blaNDM-1	Y
Patient_55	CF24	24/9/2018	2018.731507	Citrobacter freundii	blaNDM-1	Y (keep to allow pairing#)

(#) Do not exclude isolates that share exactly the same date of culture - to give a chance to both isolates to pair with source isolates. Once pairings are established, later isolate from same source patient will be excluded, resulting in unique source-acquisition patient pairs (as stated in step B2 above).

Demo Table 3: Source-acquisition pairs that meet the criteria, i.e. for each earliest isolate per patient per CP gene (acquisition isolate), find source isolates that
(i) are from **other patients** (with isolates of the same ST cluster and CP gene allele) that have **date of culture earlier or equal to the acquisition isolate**, and
(ii) SNP count of source-acquisition pair meets the BEAST-derived mutation rate threshold.
Note: The Patient ID, Isolate ID and dates stated here are for purpose of illustration and not actual patient information.

ST cluster-CP gene	Acquisition patient	Acquisition isolate	Acquisition Date of culture	Source patient	Source isolate	Source Date of culture	SNPcount	Transmission plausible
cfreundii_CCgroup22_40 samples:blaNDM-1	Patient_40	CF27	2018.816439	Patient_48	CF25	2018.797261	0	1
	Patient_14	CF31	2018.849315	Patient_40	CF27	2018.816439	1	1
	Patient_14	CF31	2018.849315	Patient_59	CF28	2018.819178	1	1
	Patient_14	CF31	2018.849315	Patient_21	CF30	2018.846576	0	1
	Patient_14	CF31	2018.849315	Patient_48	CF25	2018.797261	1	1
	Patient_36	CF20	2020.227398	Patient_51	CF10	2019.400000	5	1
	Patient_24	CF40	2019.383562	Patient_38	CF29	2018.832877	3	1
	Patient_59	CF28	2018.819178	Patient_40	CF27	2018.816439	0	1
	Patient_59	CF28	2018.819178	Patient_48	CF25	2018.797261	0	1
	Patient_18	CF15	2019.750685	Patient_4	CF1	2019.410959	0	1
	Patient_21	CF30	2018.846576	Patient_40	CF27	2018.816439	1	1
	Patient_21	CF30	2018.846576	Patient_59	CF28	2018.819178	1	1
	Patient_21	CF30	2018.846576	Patient_48	CF25	2018.797261	1	1

* Pairs that did not meet criteria or were filtered-off by steps B1 and B2 are not shown here.

(B) Determining epidemiologic relationships for source-acquisition pairs

Now that we have the source-acquisition isolate and patient pairs, we proceed to perform comparisons to determine if there were epidemiologic overlaps between the source and acquisition patients. Epidemiologic relationships between source-acquisition isolate and patient pairs were analyzed in terms of hospital admission, discipline, procedure and address (community) contact.

The same algorithm is applied to case pairs and control pairs (for control selection, see Section C below).
The custom code files for determining epidemiologic relationships are as follows and can be obtained from https://github.com/nataschamay/cp_transmission_2021/tree/main/3_find_epiOverlap

1. find_epiOverlap_Adm.pl
2. find_epiOverlap_Discipline.pl
3. find_epiOverlap_Procedure.pl
4. find_epiOverlap_Address.pl

Pseudocode for the admission hospital comparison in find_epiOverlap_Adm.pl
(a similar algorithm is used for admission ward, discipline, procedure and address comparisons, with the necessary adaptations).

Demo Table 4 shows a summary of admission overlap for the patient pairs in Demo Table 3, based on the full admission data provided in Annex A. Demo Table 5 provides some examples of how the criteria are met for hospital direct and hospital indirect contact.

```
############################## Begin Perl pseudocode ##############################

For each Source-Acquisition isolate pair{
       Store: Source-Isolate; Source-Patient; Acquisition-Isolate; Acquisition-Patient

       Loop through entire admission data row by row {

              ### Acquisition-Patient
              if (Patient of admission data row = Acquisition-Patient){

                     #Limit Acquisition-StartDate to risk period boundary (see Figure 1)
                     if(Admission-StartDate <= Acquisition-IsolateDOC){

                            if(Admission-StartDate >= Source-IsolateDOC){
                                   Acquisition-StartDate = Admission-StartDate;
                                   (i.e. store admission start date as it is)
```

```
                    }else{
                          if(Admission-StopDate >= Source-IsolateDOC){
                                Acquisition-StartDate = Source-IsolateDOC;
                                (i.e. set admission start date as source DOC)
                          }else{
                                Acquisition-StartDate out of range
                          }
                    }
              }else{
                    Acquisition-StartDate out of range
              }


              #Limit Acquisition-StopDate to risk period boundary (see Figure 1)
              if(Admission-StopDate <= Acquisition-IsolateDOC){

                    if(Admission-StopDate >= Source-IsolateDOC){
                          Acquisition-StopDate = Admission-StopDate;
                          (i.e. store admission stop date as it is)
                    }else{
                          Acquisition-StopDate out of range
                    }
              }else{
                    if(Admission-StartDate <= Acquisition-IsolateDOC){
                          Acquisition-StopDate = Acquisition-IsolateDOC;
                          (i.e. set admission stop date as acquisition DOC)
                    }else{
                          Acquisition-StopDate out of range
                    }
              }

              # Store admission data
              if Acquisition dates are within risk period range{
                    Keep storing all admission data rows for this Acquisition-Patient
                    (Hospital, Ward, Bed, Acquisition-StartDate and Acquisition-StopDate(adjusted to risk period
boundary))
              }
        }

        ### Source-Patient
        if (Patient of admission data row = Source-Patient){

              #Limit Source-StartDate to risk period boundary (see Figure 1)
              if(Admission-StartDate >= Source-IsolateDOC){
```

```
                    if(Admission-StartDate > Acquisition-IsolateDOC){
                           Source-StartDate out of range
                    }else{
                           Source-StartDate = Admission-StartDate;
                    }
             }else{
                    if(Admission-StopDate >= Source-IsolateDOC){
                           Source-StartDate = Source-IsolateDOC;
                    }else{
                           Source-StartDate out of range
                    }
             }

             #Limit Source-StopDate to risk period boundary (see Figure 1)
             if(Admission-StopDate >= Source-IsolateDOC){

                    if(Admission-StopDate > Acquisition-IsolateDOC){
                           if(Admission-StartDate <= Acquisition-IsolateDOC){
                                  Source-StopDate = Acquisition-IsolateDOC;
                           }else{
                                  Source-StopDate out of range
                           }
                    }else{
                           Source-StopDate = Admission-StopDate;
                    }
             }else{
                    Source-StopDate out of range
             }

             # Store admission data
             if Source dates are within risk period range{
                    Keep storing all admission data rows for this Source-Patient
                    (Hospital, Ward, Bed, Source-StartDate and Source-StopDate (adjusted to risk period boundary))
             }
      }
}


# Assessing Hospital Direct or Indirect Contact - comparison of all-against-all admission rows that are relevant
       For each Acquisition-Patient admission data row{

              For each Source-Patient admission data row{
```

```
            if (If the Acquisition and Source Admission Hospital matches){

                if (Source-StartDate <= Acquisition-StopDate
                        && Source-StopDate >= Acquisition-StartDate) {

                    #  |----Source-----|
                    #             |-----Acquisition----|

                    #  |----Source-------------------------|
                    #             |-----Acquisition----|

                    #                                |----Source-----|
                    #             |-----Acquisition----|
                    # Note: Source already defined as DOC earlier than Acquisition

                    Contact = "Hospital Direct Contact"

                }elsif(Source-StopDate < Acquisition-StartDate){

                    # Source admitted before Acquisition but no overlap
                    #  |----Source----|
                    #                        |-----Acquisition----|

                    Contact = "Hospital Indirect Contact"

                }else{

                    Contact = "No Hospital Contact"; # Acquisition admitted and left before Source entered (for
THIS comparison)
                }

            }else{
                Contact = "No Hospital Contact"; # Admission Hospital not matched (for THIS comparison)
            }
        }
    }
}

############################## End Perl pseudocode ##############################
```

Demo Figure 1: Limiting admission start and stop dates to risk period boundary. This is done to simplify the processing, because admission overlaps outside of the risk period boundary are not relevant.

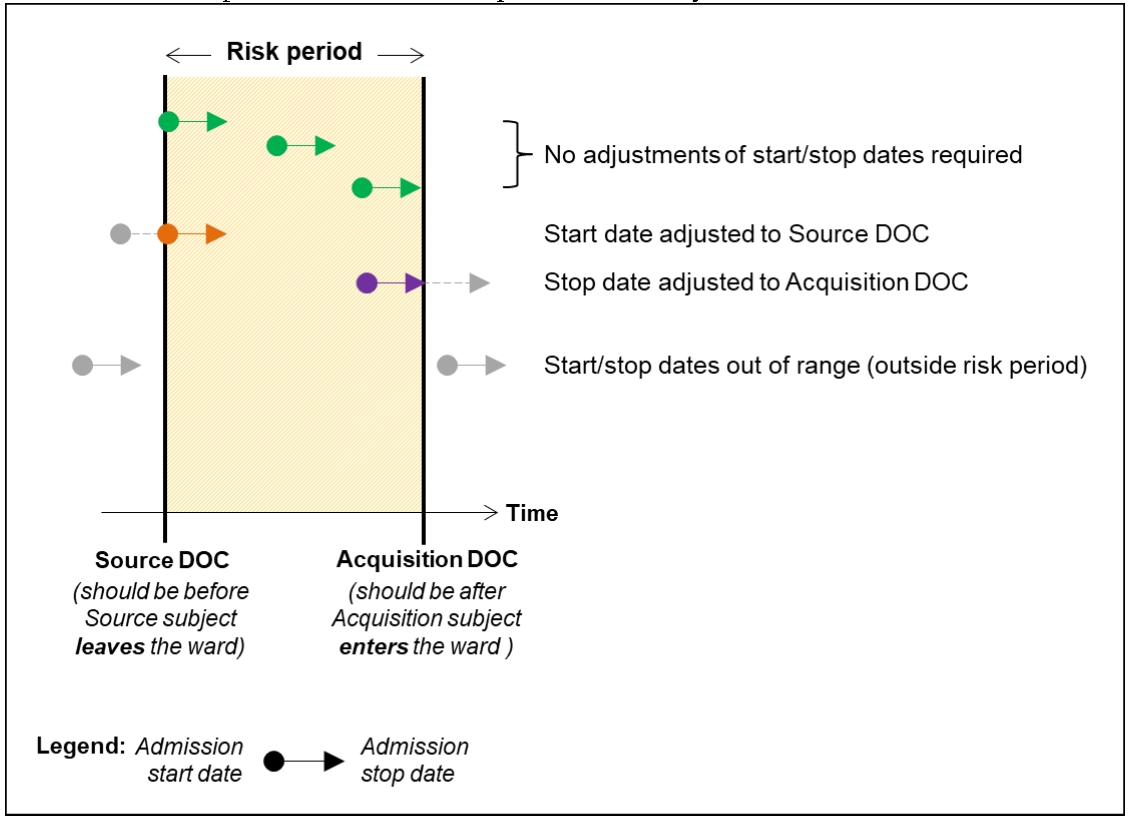

Demo Table 4: Summary of admission overlap for the patient pairs in Demo Table 3, based on the full admission data provided in Annex A.

Further details on how the criteria are met is shown in Demo Table 5 for selected outcomes highlighted in green/blue here.

Note: The Patient ID and Isolate ID stated here are for purpose of illustration and not actual patient information.

Acquisition patient	Acquisition isolate	Source patient	Source isolate	Hospital Direct	Hospital Indirect	No Hospital Contact	Ward Direct	Ward Indirect	No Ward Contact	Bed Indirect	No Bed Contact	Discipline Direct	Discipline Indirect	No Discipline Contact
Patient_40	CF27	Patient_48	CF25	Hospital Direct			Ward Direct				No Bed Contact	Discipline Direct		
Patient_14	CF31	Patient_40	CF27	Hospital Direct	Hospital Indirect				No Ward Contact		No Bed Contact			No Discipline Contact
Patient_14	CF31	Patient_59	CF28	Hospital Direct	Hospital Indirect				No Ward Contact		No Bed Contact			No Discipline Contact
Patient_14	CF31	Patient_21	CF30	Hospital Direct					No Ward Contact		No Bed Contact			No Discipline Contact
Patient_14	CF31	Patient_48	CF25	Hospital Direct	Hospital Indirect				No Ward Contact		No Bed Contact			No Discipline Contact
Patient_36	CF20	Patient_51	CF10			No Hospital Contact			No Ward Contact		No Bed Contact			No Discipline Contact
Patient_24	CF40	Patient_38	CF29		Hospital Indirect			Ward Indirect			No Bed Contact		Discipline Indirect	
Patient_59	CF28	Patient_40	CF27	Hospital Direct					No Ward Contact		No Bed Contact	Discipline Direct		
Patient_59	CF28	Patient_48	CF25	Hospital Direct			Ward Direct				No Bed Contact	Discipline Direct		
Patient_18	CF15	Patient_4	CF1	Hospital Direct	Hospital Indirect		Ward Direct	Ward Indirect			No Bed Contact	Discipline Direct	Discipline Indirect	
Patient_21	CF30	Patient_40	CF27	Hospital Direct	Hospital Indirect				No Ward Contact		No Bed Contact	Discipline Direct	Discipline Indirect	
Patient_21	CF30	Patient_59	CF28	Hospital Direct					No Ward Contact		No Bed Contact	Discipline Direct		
Patient_21	CF30	Patient_48	CF25	Hospital Direct			Ward Direct				No Bed Contact	Discipline Direct		

Demo Table 5: Examples of how the criteria are met for hospital direct and hospital indirect contact (similarly highlighted green/blue here to match Demo Table 4). *Note: The Patient ID, Isolate ID and dates stated here are for purpose of illustration and not actual patient information.*

Acquisition patient	Acquisition isolate	Acquisition Date of Culture (DOC)	Source patient	Source isolate	Source Date of Culture (DOC)	Acquisition admission start date	Acquisition admission stop date	Source admission start date	Source admission stop date	Criteria met to determine contact category	Contact category	Remarks
Patient_24	CF40	2019.383562	Patient_38	CF29	2018.832877	2019.375343	2019.383562	2018.846576	2018.868493	2018.868493 < 2019.375343	Hospital Indirect	Source admission stop date < Acquisition admission start date; Dates are already within risk period (between Source DOC and Acquisition DOC)
Patient_24	CF40	2019.383562	Patient_38	CF29	2018.832877	2019.375343	2019.383562	2018.950685	2018.953425	2018.953425 < 2019.375343	Hospital Indirect	
Patient_24	CF40	2019.383562	Patient_38	CF29	2018.832877	2019.375343	2019.383562	2018.950685	2018.953425	2018.953425 < 2019.375343	Hospital Indirect	
Patient_24	CF40	2019.383562	Patient_38	CF29	2018.832877	2019.375343	2019.383562	2018.80548	2018.835617	2018.835617 < 2019.375343	Hospital Indirect	
Patient_24	CF40	2019.383562	Patient_38	CF29	2018.832877	2019.375343	2019.383562	2018.835617	2018.841096	2018.841096 < 2019.375343	Hospital Indirect	
Patient_24	CF40	2019.383562	Patient_38	CF29	2018.832877	2019.375343	2019.383562	2018.846576	2018.868493	2018.868493 < 2019.375343	Hospital Indirect	
Patient_24	CF40	2019.383562	Patient_38	CF29	2018.832877	2019.375343	2019.383562	2018.835617	2018.841096	2018.841096 < 2019.375343	Hospital Indirect	
Patient_24	CF40	2019.383562	Patient_38	CF29	2018.832877	2019.375343	2019.383562	2018.80548	2018.835617	2018.835617 < 2019.375343	Hospital Indirect	
Patient_24	CF40	2019.383562	Patient_38	CF29	2018.832877	2019.375343	2019.383562	2018.841096	2018.846576	2018.846576 < 2019.375343	Hospital Indirect	
Patient_24	CF40	2019.383562	Patient_38	CF29	2018.832877	2019.375343	2019.383562	2018.841096	2018.846576	2018.846576 < 2019.375343	Hospital Indirect	
Patient_24	CF40	2019.383562	Patient_38	CF29	2018.832877	2019.375343	2019.383562	2018.953425	2018.953425	2018.953425 < 2019.375343	Hospital Indirect	
Patient_24	CF40	2019.383562	Patient_38	CF29	2018.832877	2019.375343	2019.383562	2018.953425	2018.953425	2018.953425 < 2019.375343	Hospital Indirect	
Patient_24	CF40	2019.383562	Patient_38	CF29	2018.832877	2019.383562	2019.386302	2018.846576	2018.868493	2018.868493 < 2019.383562	Hospital Indirect	
Patient_24	CF40	2019.383562	Patient_38	CF29	2018.832877	2019.383562	2019.386302	2018.950685	2018.953425	2018.953425 < 2019.383562	Hospital Indirect	
Patient_24	CF40	2019.383562	Patient_38	CF29	2018.832877	2019.383562	2019.386302	2018.950685	2018.953425	2018.953425 < 2019.383562	Hospital Indirect	
Patient_24	CF40	2019.383562	Patient_38	CF29	2018.832877	2019.383562	2019.386302	2018.80548	2018.835617	2018.835617 < 2019.383562	Hospital Indirect	
Patient_24	CF40	2019.383562	Patient_38	CF29	2018.832877	2019.383562	2019.386302	2018.835617	2018.841096	2018.841096 < 2019.383562	Hospital Indirect	
Patient_24	CF40	2019.383562	Patient_38	CF29	2018.832877	2019.383562	2019.386302	2018.846576	2018.868493	2018.868493 < 2019.383562	Hospital Indirect	
Patient_24	CF40	2019.383562	Patient_38	CF29	2018.832877	2019.383562	2019.386302	2018.835617	2018.841096	2018.841096 < 2019.383562	Hospital Indirect	
Patient_24	CF40	2019.383562	Patient_38	CF29	2018.832877	2019.383562	2019.386302	2018.80548	2018.835617	2018.835617 < 2019.383562	Hospital Indirect	
Patient_24	CF40	2019.383562	Patient_38	CF29	2018.832877	2019.383562	2019.386302	2018.841096	2018.846576	2018.846576 < 2019.383562	Hospital Indirect	
Patient_24	CF40	2019.383562	Patient_38	CF29	2018.832877	2019.383562	2019.386302	2018.841096	2018.846576	2018.846576 < 2019.383562	Hospital Indirect	
Patient_24	CF40	2019.383562	Patient_38	CF29	2018.832877	2019.383562	2019.386302	2018.953425	2018.953425	2018.953425 < 2019.383562	Hospital Indirect	
Patient_24	CF40	2019.383562	Patient_38	CF29	2018.832877	2019.383562	2019.386302	2018.953425	2018.953425	2018.953425 < 2019.383562	Hospital Indirect	

Patient_24	CF40	2019.383562	Patient_38	CF29	2018.832877	2019.383562	2019.386302	2018.846576	2018.868493	2018.868493 < 2019.383562	Hospital Indirect	
Patient_24	CF40	2019.383562	Patient_38	CF29	2018.832877	2019.383562	2019.386302	2018.950685	2018.953425	2018.953425 < 2019.383562	Hospital Indirect	
Patient_24	CF40	2019.383562	Patient_38	CF29	2018.832877	2019.383562	2019.386302	2018.950685	2018.953425	2018.953425 < 2019.383562	Hospital Indirect	
Patient_24	CF40	2019.383562	Patient_38	CF29	2018.832877	2019.383562	2019.386302	2018.80548	2018.835617	2018.835617 < 2019.383562	Hospital Indirect	
Patient_24	CF40	2019.383562	Patient_38	CF29	2018.832877	2019.383562	2019.386302	2018.835617	2018.841096	2018.841096 < 2019.383562	Hospital Indirect	
Patient_24	CF40	2019.383562	Patient_38	CF29	2018.832877	2019.383562	2019.386302	2018.846576	2018.868493	2018.868493 < 2019.383562	Hospital Indirect	
Patient_24	CF40	2019.383562	Patient_38	CF29	2018.832877	2019.383562	2019.386302	2018.835617	2018.841096	2018.841096 < 2019.383562	Hospital Indirect	
Patient_24	CF40	2019.383562	Patient_38	CF29	2018.832877	2019.383562	2019.386302	2018.80548	2018.835617	2018.835617 < 2019.383562	Hospital Indirect	
Patient_24	CF40	2019.383562	Patient_38	CF29	2018.832877	2019.383562	2019.386302	2018.841096	2018.846576	2018.846576 < 2019.383562	Hospital Indirect	
Patient_24	CF40	2019.383562	Patient_38	CF29	2018.832877	2019.383562	2019.386302	2018.841096	2018.846576	2018.846576 < 2019.383562	Hospital Indirect	
Patient_24	CF40	2019.383562	Patient_38	CF29	2018.832877	2019.383562	2019.386302	2018.953425	2018.953425	2018.953425 < 2019.383562	Hospital Indirect	
Patient_24	CF40	2019.383562	Patient_38	CF29	2018.832877	2019.383562	2019.386302	2018.953425	2018.953425	2018.953425 < 2019.383562	Hospital Indirect	
Patient_24	CF40	2019.383562	Patient_38	CF29	2018.832877	2019.375343	2019.383562	2018.846576	2018.868493	2018.868493 < 2019.375343	Hospital Indirect	
Patient_24	CF40	2019.383562	Patient_38	CF29	2018.832877	2019.375343	2019.383562	2018.950685	2018.953425	2018.953425 < 2019.375343	Hospital Indirect	
Patient_24	CF40	2019.383562	Patient_38	CF29	2018.832877	2019.375343	2019.383562	2018.950685	2018.953425	2018.953425 < 2019.375343	Hospital Indirect	
Patient_24	CF40	2019.383562	Patient_38	CF29	2018.832877	2019.375343	2019.383562	2018.80548	2018.835617	2018.835617 < 2019.375343	Hospital Indirect	
Patient_24	CF40	2019.383562	Patient_38	CF29	2018.832877	2019.375343	2019.383562	2018.835617	2018.841096	2018.841096 < 2019.375343	Hospital Indirect	
Patient_24	CF40	2019.383562	Patient_38	CF29	2018.832877	2019.375343	2019.383562	2018.846576	2018.868493	2018.868493 < 2019.375343	Hospital Indirect	
Patient_24	CF40	2019.383562	Patient_38	CF29	2018.832877	2019.375343	2019.383562	2018.835617	2018.841096	2018.841096 < 2019.375343	Hospital Indirect	
Patient_24	CF40	2019.383562	Patient_38	CF29	2018.832877	2019.375343	2019.383562	2018.80548	2018.835617	2018.835617 < 2019.375343	Hospital Indirect	
Patient_24	CF40	2019.383562	Patient_38	CF29	2018.832877	2019.375343	2019.383562	2018.841096	2018.846576	2018.846576 < 2019.375343	Hospital Indirect	
Patient_24	CF40	2019.383562	Patient_38	CF29	2018.832877	2019.375343	2019.383562	2018.841096	2018.846576	2018.846576 < 2019.375343	Hospital Indirect	
Patient_24	CF40	2019.383562	Patient_38	CF29	2018.832877	2019.375343	2019.383562	2018.953425	2018.953425	2018.953425 < 2019.375343	Hospital Indirect	
Patient_24	CF40	2019.383562	Patient_38	CF29	2018.832877	2019.375343	2019.383562	2018.953425	2018.953425	2018.953425 < 2019.375343	Hospital Indirect	
Patient_21	CF30	2018.846576	Patient_40	CF27	2018.816439	2018.821918	2018.852055	2018.813699	2018.816439	2018.816439 < 2018.821918	Hospital Indirect	
Patient_21	CF30	2018.846576	Patient_40	CF27	2018.816439	2018.821918	2018.852055	2018.816439	2018.819178	2018.819178 < 2018.821918	Hospital Indirect	
Patient_21	CF30	2018.846576	Patient_40	CF27	2018.816439	2018.821918	2018.852055	2018.827398	2018.835617	2018.827398 <= 2018.846576 && 2018.835617 >= 2018.821918	Hospital Direct	Source admission start date <= Acquisition admission stop date && Source admission stop date >= Acquisition admission start date; (Acquisition admission stop date limited to risk
Patient_21	CF30	2018.846576	Patient_40	CF27	2018.816439	2018.821918	2018.852055	2018.819178	2018.827398	2018.819178 <= 2018.846576 && 2018.827398 >= 2018.821918	Hospital Direct	

Patient_21	CF30	2018.846576	Patient_59	CF28	2018.819178	2018.821918	2018.852055	2018.827398	2018.830137	2018.827398 <= 2018.846576 && 2018.830137 >= 2018.821918	Hospital Direct	period; i.e. Acquisition DOC 2018.846576)
Patient_21	CF30	2018.846576	Patient_59	CF28	2018.819178	2018.821918	2018.852055	2018.816439	2018.827398	2018.819178 <= 2018.846576 && 2018.827398 >= 2018.821918	Hospital Direct	Source admission start date <= Acquisition admission stop date# && Source admission stop date >= Acquisition admission start date (Source admission start date & Acquisition admission stop date limited to risk period, i.e. Acquisition DOC 2018.846576; Source DOC 2018.819178)

(C) Selection of controls

For control pair selection, the same source patient and isolate as the case pair was retained in the control pair. The section below explains how random selection was performed to identify the control acquisition patient and isolate.

1. For a given **clonal transmission** case pair, the control acquisition patient must have
   - Different patient from the case acquisition patient
   - Different patient from the case source patient
   - Date of culture same day or after the case acquisition patient
   - The control acquisition patient must **not** meet clonal transmission criteria with the source or acquisition patient in the case pair

2. For a given **plasmid-mediated transmission** case pair,
   - Different patient from the case acquisition patient
   - Different patient from the case source patient
   - Date of culture same day or after the case acquisition patient
   - The control acquisition patient must not meet clonal transmission criteria with the source or acquisition patient in the case pair
   - The control acquisition patient must also **not** meet the plasmid-mediated transmission criteria with the source or acquisition patient in the case pair

The controls were selected independent of species, CP gene and exposure information (hospital, ward, discipline, procedure or community contact).

The custom code files for this step are **find_controls_part1.pl** and **find_controls_part2.pl**, and can be obtained from https://github.com/nataschamay/cp_transmission_2021/tree/main/4_find_controls

Pseudocode for find_controls_part1.pl for clonal transmission case pairs
(a similar code was used for plasmid-mediated transmission case pairs, with the necessary adaptations to the criteria).

```
For EACH acquisition isolate that has been established to be linked by clonal transmission{

       Loop through ALL other patients that are NOT linked by clonal transmission{

              if (the "other" patient meets all the following criteria
                     (1) Different patient from the acquisition patient,
                     (2) Different patient from all the source patients linked by clonal transmission to the acquisition patient
                            (source patients were stored in a hash-of-arrays "acquisition patients => [array of source patients]"
                            to enable this check), and
                     (3) Date of culture same day or after the acquisition patient)

                     {
                            Output the acquisition isolate and the valid control patient to a file

                     }
       }
}
```

The above method would result in a file that identifies all possible control acquisition patients which correspond to a given case pair. As stated above, the control source patient is identical to the source patient in the case pair.

This list of candidate controls for each acquisition patient is processed manually (using Microsoft Excel) to:

1. sort by Acquisition_PatientID and Control_PatientID,
2. remove duplicate control isolates from the same patient,
3. remove control isolates of patients that lack admission data,

Subsequently, the list is processed as follows:

Pseudocode for find_controls_part2.pl for clonal transmission case pairs
(a similar code was used for plasmid-mediated transmission case pairs, with the necessary adaptations).

```
For EACH acquisition-source pair{

       For EACH acquisition-candidate control pair{

              if (control isolate is NOT linked by clonal transmission to the source isolate){
                     Accept the control as valid and store in an array
              }
```

```
        }

        Randomly select a valid control for this acquisition patient
        (by using a random number generator to determine the index of the array of valid controls to be selected)
}
```

Demo Table 6: Control selection for Source-Acquisition case pairs established in Demo Table 3 above

Acquisition patient	Acquisition isolate	Acquisition DOC	Acquisition Species	Acquisition CP gene	Source patient	Source isolate	Control patient	Control isolate	Control DOC	Control Species	Control CP gene	Patient check	Date check
Patient_40	CF27	2018.816439	Citrobacter freundii	blaNDM-1	Patient_48	CF25	Patient_62	CR1	2019.326028	Citrobacter rodentium	blaNDM-1	Control isolate is from a different patient compared to Acquisition and Source isolates	Control isolate date of culture is the same or later than Acquisition isolate
Patient_14	CF31	2018.849315	Citrobacter freundii	blaNDM-1	Patient_40	CF27	Patient_63	ECO5	2019.80548	Escherichia coli	blaKPC-2		
Patient_14	CF31	2018.849315	Citrobacter freundii	blaNDM-1	Patient_59	CF28	Patient_16	CF14	2019.70685	Citrobacter freundii	blaNDM-1		
Patient_14	CF31	2018.849315	Citrobacter freundii	blaNDM-1	Patient_21	CF30	Patient_64	KP14	2019.476713	Klebsiella pneumoniae	blaOXA-48		
Patient_14	CF31	2018.849315	Citrobacter freundii	blaNDM-1	Patient_48	CF25	Patient_62	CR1	2019.326028	Citrobacter rodentium	blaNDM-1		
Patient_36	CF20	2020.227398	Citrobacter freundii	blaNDM-1	Patient_51	CF10	Patient_66	ECL43	2020.232877	Enterobacter cloacae	blaIMI-1		
Patient_24	CF40	2019.383562	Citrobacter freundii	blaNDM-1, blaOXA-48	Patient_38	CF29	Patient_67	KP15	2019.597261	Klebsiella pneumoniae	blaNDM-1		
Patient_59	CF28	2018.819178	Citrobacter freundii	blaNDM-1	Patient_40	CF27	Patient_6	ECO1	2019.446576	Escherichia coli	blaNDM-1		
Patient_59	CF28	2018.819178	Citrobacter freundii	blaNDM-1	Patient_48	CF25	Patient_68	ECO7	2019.142466	Escherichia coli	blaNDM-1		
Patient_18	CF15	2019.750685	Citrobacter freundii	blaNDM-1	Patient_4	CF1	Patient_69	ECO8	2020.180822	Escherichia coli	blaKPC-2		
Patient_21	CF30	2018.846576	Citrobacter freundii	blaNDM-1	Patient_40	CF27	Patient_70	KP16	2019.60274	Klebsiella pneumoniae	blaIMP-1		
Patient_21	CF30	2018.846576	Citrobacter freundii	blaNDM-1	Patient_59	CF28	Patient_71	KP17	2019.397261	Klebsiella pneumoniae	blaKPC-2		
Patient_21	CF30	2018.846576	Citrobacter freundii	blaNDM-1	Patient_48	CF25	Patient_72	KP18	2019.443836	Klebsiella pneumoniae	blaKPC-2		

Annex A: Full admission data for selected patients relevant to examples used above.

Note: The Patient ID, locations and dates stated here are for purpose of illustration and not actual patient information.

Patient_ID	Admission Hospital	Admission Ward	Discipline	Admission Start Date	Admission Stop Date
Patient_4	Hospital_A	Ward_17	general medicine	2015.063014	2015.073973
Patient_4	Hospital_A	Ward_17	general medicine	2015.073973	2015.073973
Patient_4	Hospital_A	Ward_22	general medicine	2015.10137	2015.136987
Patient_4	Hospital_A	Ward_10	general medicine	2015.150685	2015.156165
Patient_4	Hospital_A	Ward_22	general medicine	2015.156165	2015.161644
Patient_4	Hospital_A	Ward_43	cardiology	2015.210959	2015.227398
Patient_4	Hospital_A	Ward_43	cardiology	2015.252055	2015.268493
Patient_4	Hospital_A	Ward_18	general medicine	2015.342466	2015.347945
Patient_4	Hospital_A	Ward_17	general medicine	2015.347945	2015.347945
Patient_4	Hospital_A	Ward_17	nephrology	2015.473973	2015.482192
Patient_4	Hospital_A	Ward_11	nephrology	2015.482192	2015.512329
Patient_4	Hospital_A	Ward_11	nephrology	2015.545206	2015.556165
Patient_4	Hospital_A	Ward_11	nephrology	2015.556165	2015.572603
Patient_4	Hospital_A	Ward_30	general surgery	2015.638356	2015.638356
Patient_4	Hospital_A	Ward_10	nephrology	2015.742466	2015.745206
Patient_4	Hospital_C	Ward_44	Ortho / Gen Ortho	2015.816439	2015.821918
Patient_4	Hospital_A	Ward_18	general surgery	2015.863014	2015.868493
Patient_4	Hospital_A	Ward_3	general surgery	2015.958904	2015.961644
Patient_4	Hospital_C	Ward_44	Ortho / Gen Ortho	2015.986302	2015.991781
Patient_4	Hospital_C	Ward_45	Surg / Gen Surg	2016.068493	2016.071233
Patient_4	Hospital_A	Ward_2	general medicine	2016.109589	2016.126028
Patient_4	Hospital_A	Ward_6	general surgery	2016.249315	2016.257535
Patient_4	Hospital_A	Ward_10	general medicine	2016.312329	2016.315069
Patient_4	Hospital_A	Ward_18	general medicine	2016.315069	2016.331507
Patient_4	Hospital_A	Ward_13	general surgery	2016.473973	2016.476713
Patient_4	Hospital_A	Ward_3	general surgery	2016.80548	2016.808219
Patient_4	Hospital_A	Ward_3	general surgery	2016.808219	2016.810959
Patient_4	Hospital_A	Ward_19	general medicine	2016.824658	2016.824658
Patient_4	Hospital_A	Ward_22	general medicine	2016.824658	2016.843836
Patient_4	Hospital_A	Ward_6	general surgery	2016.986302	2016.994521
Patient_4	Hospital_A	Ward_6	general surgery	2017.407112	2017.412576
Patient_4	Hospital_A	Ward_18	endocrinology	2017.598368	2017.603833
Patient_4	Hospital_A	Ward_6	endocrinology	2017.603833	2017.606565
Patient_4	Hospital_A	Ward_22	endocrinology	2017.606565	2017.625691
Patient_4	Hospital_A	Ward_13	plastic, reconstruction & aesthetic surgery	2017.732248	2017.781429
Patient_4	Hospital_A	Ward_22	general medicine	2017.885254	2017.887986
Patient_4	Hospital_A	Ward_36	orthopaedics	2018.049315	2018.052055
Patient_4	Hospital_A	Ward_36	orthopaedics	2018.052055	2018.054795
Patient_4	Hospital_A	Ward_36	orthopaedics	2018.054795	2018.071233
Patient_4	Hospital_A	Ward_36	orthopaedics	2018.071233	2018.079452
Patient_4	Hospital_A	Ward_29	nephrology	2018.29863	2018.30411
Patient_4	Hospital_A	Ward_11	nephrology	2018.331507	2018.336987
Patient_4	Hospital_C	Ward_46	Med / Gen Med	2018.454795	2018.457535
Patient_4	Hospital_C	Ward_46	Med / Gen Med	2018.457535	2018.457535
Patient_4	Hospital_C	Ward_46	Med / Gen Med	2018.457535	2018.463014
Patient_4	Hospital_A	Ward_36	general medicine	2018.465754	2018.479452
Patient_4	Hospital_A	Ward_22	general medicine	2018.479452	2018.526028
Patient_4	Hospital_A	Ward_26	urology	2018.558904	2018.586302

Patient_4	Hospital_A	Ward_12	urology	2018.586302	2018.693151
Patient_4	Hospital_A	Ward_47	neurosurgery	2018.750685	2018.764384
Patient_4	Hospital_A	Ward_48	neurosurgery	2018.764384	2018.783562
Patient_4	Hospital_A	Ward_48	neurosurgery	2018.783562	2018.8
Patient_4	Hospital_A	Ward_47	neurosurgery	2018.8	2018.810959
Patient_4	Hospital_A	Ward_48	neurosurgery	2018.810959	2018.931507
Patient_4	Hospital_A	Ward_3	general surgery	2019.09863	2019.128767
Patient_4	Hospital_A	Ward_11	nephrology	2019.134247	2019.147945
Patient_4	Hospital_A	Ward_11	nephrology	2019.147945	2019.147945
Patient_4	Hospital_A	Ward_11	nephrology	2019.147945	2019.175343
Patient_4	Hospital_A	Ward_22	general medicine	2019.180822	2019.183562
Patient_4	Hospital_A	Ward_48	vascular surgery	2019.20274	2019.238356
Patient_4	Hospital_A	Ward_5	vascular surgery	2019.238356	2019.265754
Patient_4	Hospital_A	Ward_17	general surgery	2019.293151	2019.323288
Patient_4	Hospital_A	Ward_3	general surgery	2019.323288	2019.410959
Patient_4	Hospital_A	Ward_6	general surgery	2019.410959	2019.413699
Patient_4	Hospital_A	Ward_15	general surgery	2019.413699	2019.443836
Patient_4	Hospital_A	Ward_27	general surgery	2019.443836	2019.512329
Patient_4	Hospital_A	Ward_15	general surgery	2019.512329	2019.515069
Patient_14	Hospital_A	Ward_1	general medicine	2017.180336	2017.1858
Patient_14	Hospital_A	Ward_2	general surgery	2018.147945	2018.156165
Patient_14	Hospital_A	Ward_3	general surgery	2018.156165	2018.158904
Patient_14	Hospital_A	Ward_4	general medicine	2018.734247	2018.736987
Patient_14	Hospital_A	Ward_2	hpb	2018.827398	2018.843836
Patient_14	Hospital_A	Ward_3	hpb	2018.843836	2018.852055
Patient_14	Hospital_A	Ward_3	hpb	2018.852055	2018.868493
Patient_18	Hospital_A	Ward_5	general surgery	2016.320548	2016.350685
Patient_18	Hospital_A	Ward_6	general surgery	2016.509589	2016.515069
Patient_18	Hospital_A	Ward_6	general surgery	2016.515069	2016.547945
Patient_18	Hospital_B	Ward_7	pulmonology	2017.172139	2017.1858
Patient_18	Hospital_B	Ward_8	GE & Hep	2017.45356	2017.459024
Patient_18	Hospital_B	Ward_9	GE & Hep	2017.459024	2017.475418
Patient_18	Hospital_A	Ward_3	general surgery	2017.814215	2017.81968
Patient_18	Hospital_A	Ward_10	general medicine	2017.86886	2017.877057
Patient_18	Hospital_A	Ward_11	general medicine	2018.065754	2018.082192
Patient_18	Hospital_A	Ward_3	general surgery	2018.260274	2018.263014
Patient_18	Hospital_A	Ward_3	general surgery	2018.263014	2018.284932
Patient_18	Hospital_A	Ward_3	general surgery	2019.10685	2019.145206
Patient_18	Hospital_A	Ward_12	general surgery	2019.221918	2019.227398
Patient_18	Hospital_A	Ward_5	general surgery	2019.227398	2019.230137
Patient_18	Hospital_A	Ward_5	general surgery	2019.230137	2019.230137
Patient_18	Hospital_A	Ward_5	general surgery	2019.230137	2019.30137
Patient_18	Hospital_A	Ward_5	general surgery	2019.30137	2019.353425
Patient_18	Hospital_A	Ward_6	general surgery	2019.375343	2019.394521
Patient_18	Hospital_A	Ward_3	general surgery	2019.394521	2019.397261
Patient_18	Hospital_A	Ward_3	general surgery	2019.397261	2019.454795
Patient_18	Hospital_A	Ward_13	general surgery	2019.509589	2019.512329
Patient_18	Hospital_A	Ward_6	general surgery	2019.512329	2019.575343
Patient_18	Hospital_A	Ward_14	vascular surgery	2019.786302	2019.810959
Patient_18	Hospital_A	Ward_15	vascular surgery	2019.810959	2019.816439
Patient_18	Hospital_A	Ward_14	vascular surgery	2019.816439	2019.884932
Patient_18	Hospital_A	Ward_14	vascular surgery	2019.884932	2019.890411
Patient_21	Hospital_A	Ward_16	neurology	2016.671233	2016.679452

Patient_21	Hospital_A	Ward_16	neurology	2016.679452	2016.70411
Patient_21	Hospital_A	Ward_17	general medicine	2018.797261	2018.80548
Patient_21	Hospital_A	Ward_18	general medicine	2018.821918	2018.852055
Patient_21	Hospital_A	Ward_15	general medicine	2018.945206	2018.953425
Patient_24	Hospital_A	Ward_6	general surgery	2019.375343	2019.383562
Patient_24	Hospital_A	Ward_6	general surgery	2019.383562	2019.386302
Patient_24	Hospital_A	Ward_6	general surgery	2019.386302	2019.391781
Patient_24	Hospital_A	Ward_3	general surgery	2019.438356	2019.449315
Patient_24	Hospital_A	Ward_3	general surgery	2019.449315	2019.463014
Patient_24	Hospital_A	Ward_6	general surgery	2019.463014	2019.471233
Patient_24	Hospital_A	Ward_15	general surgery	2019.471233	2019.50411
Patient_24	Hospital_A	Ward_15	general surgery	2019.50411	2019.553425
Patient_24	Hospital_A	Ward_6	general surgery	2019.375343	2019.383562
Patient_24	Hospital_A	Ward_6	general surgery	2019.383562	2019.386302
Patient_24	Hospital_A	Ward_6	general surgery	2019.386302	2019.391781
Patient_24	Hospital_A	Ward_3	general surgery	2019.438356	2019.449315
Patient_24	Hospital_A	Ward_3	general surgery	2019.449315	2019.463014
Patient_24	Hospital_A	Ward_6	general surgery	2019.463014	2019.471233
Patient_24	Hospital_A	Ward_15	general surgery	2019.471233	2019.50411
Patient_24	Hospital_A	Ward_15	general surgery	2019.50411	2019.553425
Patient_36	Hospital_A	Ward_19	general surgery	2019.312329	2019.317808
Patient_36	Hospital_A	Ward_20	haematology	2019.353425	2019.378082
Patient_36	Hospital_A	Ward_20	haematology	2019.378082	2019.40548
Patient_36	Hospital_A	Ward_21	haematology	2019.424658	2019.430137
Patient_36	Hospital_A	Ward_20	haematology	2019.454795	2019.457535
Patient_36	Hospital_A	Ward_22	haematology	2019.843836	2019.849315
Patient_36	Hospital_A	Ward_20	haematology	2019.849315	2019.865754
Patient_36	Hospital_A	Ward_18	haematology	2019.879452	2019.879452
Patient_36	Hospital_A	Ward_23	haematology	2019.879452	2019.893151
Patient_36	Hospital_A	Ward_20	haematology	2019.909589	2019.917808
Patient_36	Hospital_A	Ward_20	haematology	2019.964384	2019.994521
Patient_36	Hospital_A	Ward_24	haematology	2020.008219	2020.010959
Patient_36	Hospital_A	Ward_20	haematology	2020.010959	2020.013699
Patient_36	Hospital_A	Ward_20	haematology	2020.013699	2020.019178
Patient_36	Hospital_A	Ward_20	haematology	2020.038356	2020.054795
Patient_36	Hospital_A	Ward_21	haematology	2020.073973	2020.084932
Patient_36	Hospital_A	Ward_20	haematology	2020.084932	2020.093151
Patient_36	Hospital_A	Ward_20	haematology	2020.10137	2020.131507
Patient_36	Hospital_A	Ward_20	haematology	2020.131507	2020.136987
Patient_36	Hospital_A	Ward_23	haematology	2020.158904	2020.2
Patient_36	Hospital_A	Ward_20	haematology	2020.227398	2020.235617
Patient_36	Hospital_A	Ward_15	haematology	2020.235617	2020.249315
Patient_36	Hospital_A	Ward_23	haematology	2020.284932	2020.287672
Patient_36	Hospital_A	Ward_15	haematology	2020.287672	2020.30137
Patient_36	Hospital_A	Ward_15	haematology	2020.312329	2020.315069
Patient_36	Hospital_A	Ward_15	haematology	2020.315069	2020.350685
Patient_36	Hospital_A	Ward_24	haematology	2020.389041	2020.391781
Patient_36	Hospital_A	Ward_15	haematology	2020.40274	2020.410959
Patient_38	Hospital_A	Ward_25	general surgery	2018.158904	2018.164384
Patient_38	Hospital_A	Ward_6	general surgery	2018.80548	2018.835617
Patient_38	Hospital_A	Ward_3	general surgery	2018.835617	2018.841096
Patient_38	Hospital_A	Ward_3	general surgery	2018.841096	2018.846576
Patient_38	Hospital_A	Ward_3	general surgery	2018.846576	2018.868493

Patient_38	Hospital_A	Ward_2	pulmonology	2018.950685	2018.953425
Patient_38	Hospital_A	Ward_14	pulmonology	2018.953425	2018.953425
Patient_38	Hospital_A	Ward_3	vascular surgery	2019.520548	2019.523288
Patient_38	Hospital_A	Ward_15	vascular surgery	2019.523288	2019.526028
Patient_38	Hospital_A	Ward_26	urology	2019.663014	2019.665754
Patient_38	Hospital_A	Ward_14	urology	2019.665754	2019.668493
Patient_38	Hospital_A	Ward_14	oncology	2019.734247	2019.830137
Patient_38	Hospital_A	Ward_6	general surgery	2018.80548	2018.835617
Patient_38	Hospital_A	Ward_3	general surgery	2018.835617	2018.841096
Patient_38	Hospital_A	Ward_3	general surgery	2018.841096	2018.846576
Patient_38	Hospital_A	Ward_3	general surgery	2018.846576	2018.868493
Patient_38	Hospital_A	Ward_2	pulmonology	2018.950685	2018.953425
Patient_38	Hospital_A	Ward_14	pulmonology	2018.953425	2018.953425
Patient_38	Hospital_A	Ward_3	vascular surgery	2019.520548	2019.523288
Patient_38	Hospital_A	Ward_15	vascular surgery	2019.523288	2019.526028
Patient_38	Hospital_A	Ward_26	urology	2019.663014	2019.665754
Patient_38	Hospital_A	Ward_14	urology	2019.665754	2019.668493
Patient_38	Hospital_A	Ward_14	oncology	2019.734247	2019.830137
Patient_40	Hospital_A	Ward_17	general medicine	2018.791781	2018.794521
Patient_40	Hospital_A	Ward_17	general medicine	2018.794521	2018.813699
Patient_40	Hospital_A	Ward_27	general medicine	2018.813699	2018.816439
Patient_40	Hospital_A	Ward_28	general medicine	2018.816439	2018.819178
Patient_40	Hospital_A	Ward_29	general medicine	2018.819178	2018.827398
Patient_40	Hospital_A	Ward_15	general medicine	2018.827398	2018.835617
Patient_40	Hospital_A	Ward_15	general surgery	2018.868493	2018.939726
Patient_40	Hospital_A	Ward_21	oncology	2018.961644	2018.972603
Patient_40	Hospital_A	Ward_15	oncology	2018.972603	2018.980822
Patient_40	Hospital_A	Ward_15	oncology	2019.019178	2019.046576
Patient_40	Hospital_A	Ward_15	oncology	2019.046576	2019.076713
Patient_40	Hospital_A	Ward_15	oncology	2019.095891	2019.136987
Patient_40	Hospital_A	Ward_15	oncology	2019.172603	2019.194521
Patient_40	Hospital_A	Ward_27	oncology	2019.194521	2019.197261
Patient_48	Hospital_A	Ward_29	nephrology	2015.523288	2015.534247
Patient_48	Hospital_A	Ward_29	nephrology	2015.534247	2015.536987
Patient_48	Hospital_A	Ward_11	nephrology	2015.550685	2015.553425
Patient_48	Hospital_A	Ward_29	nephrology	2015.926028	2015.945206
Patient_48	Hospital_A	Ward_30	general surgery	2016	2016
Patient_48	Hospital_A	Ward_30	general surgery	2016.115069	2016.115069
Patient_48	Hospital_A	Ward_31	cardiology	2016.156165	2016.158904
Patient_48	Hospital_A	Ward_32	cardiology	2016.158904	2016.158904
Patient_48	Hospital_A	Ward_10	nephrology	2016.167124	2016.172603
Patient_48	Hospital_A	Ward_11	nephrology	2016.172603	2016.20274
Patient_48	Hospital_A	Ward_11	nephrology	2016.20274	2016.227398
Patient_48	Hospital_A	Ward_18	nephrology	2016.246576	2016.249315
Patient_48	Hospital_A	Ward_11	nephrology	2016.249315	2016.252055
Patient_48	Hospital_A	Ward_29	nephrology	2016.893151	2016.89863
Patient_48	Hospital_A	Ward_29	nephrology	2016.912329	2016.912329
Patient_48	Hospital_A	Ward_33	nephrology	2016.912329	2016.915069
Patient_48	Hospital_A	Ward_29	nephrology	2016.915069	2016.920548
Patient_48	Hospital_A	Ward_17	general medicine	2017.139352	2017.226784
Patient_48	Hospital_A	Ward_29	general medicine	2017.226784	2017.226784
Patient_48	Hospital_A	Ward_17	general medicine	2017.226784	2017.229516
Patient_48	Hospital_A	Ward_29	general medicine	2017.229516	2017.229516

Patient_48	Hospital_A	Ward_17	general medicine	2017.229516	2017.240445
Patient_48	Hospital_A	Ward_28	general medicine	2017.240445	2017.248642
Patient_48	Hospital_A	Ward_28	general medicine	2017.248642	2017.29509
Patient_48	Hospital_A	Ward_17	general medicine	2017.29509	2017.420773
Patient_48	Hospital_B	Ward_34	neurology	2018.10411	2018.10411
Patient_48	Hospital_B	Ward_35	neurology	2018.10411	2018.10685
Patient_48	Hospital_B	Ward_34	neurology	2018.10685	2018.109589
Patient_48	Hospital_B	Ward_35	neurology	2018.109589	2018.112329
Patient_48	Hospital_B	Ward_34	neurology	2018.112329	2018.131507
Patient_48	Hospital_A	Ward_6	general medicine	2018.734247	2018.739726
Patient_48	Hospital_A	Ward_28	general medicine	2018.739726	2018.750685
Patient_48	Hospital_A	Ward_17	general medicine	2018.750685	2018.821918
Patient_48	Hospital_A	Ward_17	general medicine	2018.821918	2019.013699
Patient_48	Hospital_A	Ward_29	general medicine	2019.013699	2019.013699
Patient_48	Hospital_A	Ward_17	general medicine	2019.013699	2019.372603
Patient_51	Hospital_A	Ward_30	orthopaedics	2015.556165	2015.556165
Patient_51	Hospital_A	Ward_29	nephrology	2015.742466	2015.742466
Patient_51	Hospital_A	Ward_29	nephrology	2015.742466	2015.761644
Patient_51	Hospital_A	Ward_29	nephrology	2015.8	2015.80274
Patient_51	Hospital_A	Ward_29	nephrology	2015.80274	2015.810959
Patient_51	Hospital_A	Ward_29	nephrology	2015.810959	2015.810959
Patient_51	Hospital_A	Ward_29	nephrology	2015.810959	2015.830137
Patient_51	Hospital_A	Ward_29	nephrology	2015.830137	2015.852055
Patient_51	Hospital_A	Ward_3	general surgery	2015.868493	2015.868493
Patient_51	Hospital_A	Ward_30	general surgery	2016.057535	2016.057535
Patient_51	Hospital_A	Ward_36	orthopaedics	2016.142466	2016.153425
Patient_51	Hospital_A	Ward_18	endocrinology	2017.532795	2017.546456
Patient_51	Hospital_A	Ward_11	nephrology	2017.562849	2017.57651
Patient_51	Hospital_A	Ward_30	hand surgery	2018.136987	2018.136987
Patient_51	Hospital_D	Ward_37	pulmonology	2018.432877	2018.432877
Patient_51	Hospital_D	Ward_38	pulmonology	2018.432877	2018.432877
Patient_51	Hospital_D	Ward_39	pulmonology	2018.432877	2018.443836
Patient_51	Hospital_D	Ward_40	pulmonology	2018.443836	2018.457535
Patient_51	Hospital_D	Ward_37	general medicine	2019.079452	2019.079452
Patient_51	Hospital_D	Ward_41	general medicine	2019.079452	2019.090411
Patient_51	Hospital_A	Ward_5	orthopaedics	2019.320548	2019.336987
Patient_51	Hospital_D	Ward_37	general medicine	2019.347945	2019.350685
Patient_51	Hospital_D	Ward_41	general medicine	2019.350685	2019.372603
Patient_51	Hospital_D	Ward_41	general medicine	2019.372603	2019.389041
Patient_51	Hospital_D	Ward_27	general medicine	2019.389041	2019.394521
Patient_51	Hospital_D	Ward_42	general medicine	2019.394521	2019.40548
Patient_51	Hospital_D	Ward_27	general medicine	2019.40548	2019.408219
Patient_51	Hospital_D	Ward_27	general medicine	2019.408219	2019.430137
Patient_59	Hospital_A	Ward_10	general surgery	2018.312329	2018.317808
Patient_59	Hospital_A	Ward_17	general medicine	2018.808219	2018.816439
Patient_59	Hospital_A	Ward_6	general medicine	2018.816439	2018.827398
Patient_59	Hospital_A	Ward_6	general medicine	2018.827398	2018.830137

References

1. Marimuthu, K. *et al.* Clinical and Molecular Epidemiology of Carbapenem-Resistant Enterobacteriaceae Among Adult Inpatients in Singapore. *Clinical Infectious Diseases* **64**, S68–S75 (2017).
2. Teo, J. *et al.* Molecular characterization of NDM-1 producing Enterobacteriaceae isolates in Singapore hospitals. *Western Pac Surveill Response J* **3**, 19–24 (2012).
3. Balm, M. N. D., Ngan, G., Jureen, R., Lin, R. T. P. & Teo, J. W. P. OXA-181-producing Klebsiella pneumoniae establishing in Singapore. *BMC Infectious Diseases* **13**, (2013).
4. Khong, W. X. *et al.* Tracking inter-institutional spread of NDM and identification of a novel NDM-positive plasmid, pSg1-NDM, using next-generation sequencing approaches. *J Antimicrob Chemother* **71**, 3081–3089 (2016).
5. Martin, M. Cutadapt removes adapter sequences from high-throughput sequencing reads. *EMBnet.journal* **17**, 10–12 (2011).
6. Bankevich, A. *et al.* SPAdes: a new genome assembly algorithm and its applications to single-cell sequencing. *J Comput Biol* **19**, 455–477 (2012).
7. Wick, R. R., Judd, L. M., Gorrie, C. L. & Holt, K. E. Unicycler: Resolving bacterial genome assemblies from short and long sequencing reads. *PLoS Comput Biol* **13**, e1005595 (2017).
8. Urwin, R. & Maiden, M. C. J. Multi-locus sequence typing: a tool for global epidemiology. *Trends Microbiol* **11**, 479–487 (2003).
9. Thomsen, M. C. F. *et al.* A Bacterial Analysis Platform: An Integrated System for Analysing Bacterial Whole Genome Sequencing Data for Clinical Diagnostics and Surveillance. *PLoS ONE* **11**, e0157718 (2016).
10. Wood, D. E. & Salzberg, S. L. Kraken: ultrafast metagenomic sequence classification using exact alignments. *Genome Biology* **15**, R46 (2014).
11. NCBI Beta-Lactamase Database. https://ftp.ncbi.nlm.nih.gov/pathogen/betalactamases/ (2017).
12. Inouye, M. *et al.* SRST2: Rapid genomic surveillance for public health and hospital microbiology labs. *Genome Medicine* **6**, 90 (2014).
13. Gupta, S. K. *et al.* ARG-ANNOT, a New Bioinformatic Tool To Discover Antibiotic Resistance Genes in Bacterial Genomes. *Antimicrob. Agents Chemother.* **58**, 212 (2014).
14. David, S. *et al.* Epidemic of carbapenem-resistant Klebsiella pneumoniae in Europe is driven by nosocomial spread. *Nat Microbiol* **4**, 1919–1929 (2019).
15. Seemann, T. Prokka: rapid prokaryotic genome annotation. *Bioinformatics* **30**, 2068–2069 (2014).
16. Page, A. J. *et al.* Roary: rapid large-scale prokaryote pan genome analysis. *Bioinformatics* **31**, 3691–3693 (2015).
17. Page, A. J. *et al.* SNP-sites: rapid efficient extraction of SNPs from multi-FASTA alignments. *Microb Genom* **2**, e000056 (2016).
18. Croucher, N. J. *et al.* Rapid phylogenetic analysis of large samples of recombinant bacterial whole genome sequences using Gubbins. *Nucleic Acids Res* **43**, e15 (2015).
19. Bouckaert, R. *et al.* BEAST 2.5: An advanced software platform for Bayesian evolutionary analysis. *PLoS Comput Biol* **15**, e1006650 (2019).
20. Stoesser, N. *et al.* Evolutionary History of the Global Emergence of the *Escherichia coli* Epidemic Clone ST131. *mBio* **7**, e02162-15, /mbio/7/2/e02162-15.atom (2016).
21. Bouckaert, R. R. & Drummond, A. J. bModelTest: Bayesian phylogenetic site model averaging and model comparison. *BMC Evol Biol* **17**, 42 (2017).

22. Eyre, D. W. *et al.* Short-term genome stability of serial Clostridium difficile ribotype 027 isolates in an experimental gut model and recurrent human disease. *PLoS One* **8**, e63540 (2013).
23. Roosaare, M., Puustusmaa, M., Möls, M., Vaher, M. & Remm, M. PlasmidSeeker: identification of known plasmids from bacterial whole genome sequencing reads. *PeerJ* **6**, e4588 (2018).
