# Supplementary material for: Whole genome sequencing reveals hidden transmission of carbapenemase-producing Enterobacterales
Source: Nat Commun. 2022 Jun 1;13:3052. doi: 10.1038/s41467-022-30637-5 (PMC9160272; doi:10.1038/s41467-022-30637-5)
Supplement: Supplementary file 3 — Description of Additional Supplementary Files [file 41467_2022_30637_MOESM3_ESM.pdf]

Description of Additional Supplementary Files

File Name: Supplementary Data 1
Description: Detailed listing of the isolates analysed.

File Name: Supplementary Software 1
Description: Custom code for statistical analysis.
