# Supplementary material for: Whole genome sequencing reveals hidden transmission of carbapenemase-producing Enterobacterales
Source: Nat Commun. 2022 Jun 1;13:3052. doi: 10.1038/s41467-022-30637-5 (PMC9160272; doi:10.1038/s41467-022-30637-5)
Supplement: Supplementary file 5 — Supplementary Software 1 [file 41467_2022_30637_MOESM5_ESM.zip › Statistical_programming_code/data/codebook.docx]

| V01 | Month (three letter name-two digit year) |
| --- | --- |
| V02 | Number of surveillance cultures |
| V03 | Number of bacteria-linked transmission |
| V04 | Number of plasmid-linked transmission |
| V05 | Number of unlinked transmission |
| V06 | Number of bacteria-linked CC |
| V07 | Number of plasmid-linked CC |
| V08 | Number of unlinked CC |
| V09 | Number of bacteria-linked SC |
| V10 | Number of plasmid-linked SC |
| V11 | Number of unlinked SC |
| V12 | Number of plasmid-linked transmissions that are: …hospital direct |
| V13 | …hospital indirect |
| V14 | …no geographical overlap |
| V15 | …ward direct |
| V16 | …ward indirect |
| V17 | Number of bacteria-linked transmissions that are: …hospital direct |
| V18 | …hospital indirect |
| V19 | …no geographical overlap |
| V20 | …ward direct |
| V21 | …ward indirect |
| V22 | Not updated do not use |
| … | Not updated do not use |
| V26 | Not updated do not use |
| V27 | Month (again) |
| V28 | PD: patient days? |
| V29 | Month (just month) |
| V30 | Year |
| V31 | Time index |
